# Adsorption air conditioning: a comprehensive review in desiccant materials, system progress, and recent studies on different configurations of hybrid solid desiccant air conditioning systems

**DOI:** 10.1007/s11356-023-25209-z

**Published:** 2023-01-18

**Authors:** Mohamed Abdelgaied, Mohamed A. Saber, Mohamed Mahgoub Bassuoni, Ahmed M. Khaira

**Affiliations:** Mechanical Power Engineering Department, Faculty of Engineering, Tanta University, Tanta, Egypt

**Keywords:** Desiccant materials, Desiccant air conditioning, Hybrid energy subsystems, HDH desalination system, Performance improvement

## Abstract

The desiccant air conditioning system has multiple advantages (e.g., no use of ozone-depleting refrigerants, highly efficient moisture control, easy regenerative integration) over traditional vapor-compression refrigeration systems, thus increasingly attracting more research interest. Recently, several studies have been conducted that primarily aimed to enhance the overall performance of desiccant air conditioners by innovating new desiccant materials, innovating new system configurations and improving system designs and controls, and integrating different hybrid energy sub-systems technologies. Therefore, this paper provides a comprehensive review of the studies mentioned earlier. The present comprehensive review dealt with several axes: first, an overview of the importance of using desiccant air conditioners and their operations, and performance indicators. Second, a summary statement for desiccant materials that includes: the new innovative desiccant materials and the most important composite desiccant materials. Third, detailed information on the newest innovative designs and configurations of desiccant air conditioning systems and their control systems. Fourth, a detailed statement on the most important hybrid energy sub-systems technologies integrated with desiccant air conditioners. Based on the latest developments in desiccant air conditioning systems, this study presents discussions of urgent issues and recommendations for future work that can help focus necessary efforts to find solutions to critical and pending problems, which lead to further improvements in the overall performance of desiccant air conditioners.

## Introduction

Greenhouse gas emissions and rising consumption of power resulting from the use of building cooling and heating systems are one of the most important challenges of the next decade, where energy consumption rates in HVAC systems reached 35% of energy consumption in commercial and residential buildings (Gao et al. 2021; Abad et al. 2022). Therefore, performance improvement of building cooling and heating systems is an urgent and necessary task to minimize greenhouse gas emissions and power consumption.

In recent decades, various technologies for heating and cooling systems in buildings have been invented and developed, for example, heat/enthalpy recovery, desiccant dehumidification, adsorption system, and absorption system to mitigate greenhouse gas emissions and increase energy consumption (Aliane et al. 2016; Zhou et al. 2012; Abdel-Salam and Simonson 2016; Mardiana-Idayu and Riffat 2012). A desiccant air conditioner is considered a promising alternative to a traditional vapor-compression system, as well as takes advantage of solar energy and waste heat in the operation of the desiccant air conditioners (La et al. 2010). In contrast to a vapor-compression system, a desiccant air conditioner is therefore environmentally friendly. The desiccant air conditioning system uses sorbent to remove air moisture. This method differs from the traditional method utilized in traditional vapor-compression refrigeration systems, which works to condense moisture by cooling below dew point temperature, and then the air is reheated to the required temperature. In a desiccant air conditioner, sensible cooling was achieved through various methods, for example, cooling towers, evaporative coolers, and absorption chillers.

The use of desiccant air conditioners has proven to be highly effective in terms of economic, carbon emissions, and energy performance compared to the vapor-compression systems (Qi et al. 2012; Baniyounes et al. 2013a, b;). The use of solar-assisted liquid desiccant air conditioners can reduce energy consumption rates by up to 54% and 81% for hotel buildings and office buildings, respectively, compared to traditional vapor-compression refrigeration systems (Qi et al. 2012). Baniyounes et al. (2013a, b) developed solar rotary desiccant wheel air conditioners for institutional buildings in which annual power consumption rates were reduced by 17.8% compared to a vapor-compression refrigeration system. Kim et al. (2014) provide a comparison between the behavior of liquid desiccant air conditioners and traditional vapor-compression refrigeration systems. The outcomes of the comparison showed that the use of liquid desiccant air conditioners reduces annual power consumption by 68% compared to the traditional vapor-compression refrigeration systems. Speerforck and Schmitz (2016) developed a desiccant air conditioner by incorporating it with solar collectors, which reduced electricity consumption rates by up to 70% compared to a vapor-compression system. So Liu et al. (2018) incorporated the liquid desiccant air conditioning with a vapor-compression system; the coefficient of performance of the hybrid system was achieved ranging from 5.96 to 6.68. The desiccant air conditioner has also been combined with thermoelectric coolers (Li et al. 2019), humidification-dehumidification desalination (Kabeel et al. 2017b), and refrigerant sub-cooling (She et al. 2018) to improve configurations of desiccant air conditioners and thus enhance overall system performance.

Güzelel et al. (2022) theoretically studied the influences of the mixing ratio of exhaust air and desired building air temperature on the behavior of rotary desiccant air conditioners integrated with heat recovery units and dew-point indirect evaporative coolers, and direct evaporative coolers. They concluded that the average monthly thermal COP reached a maximum value of 0.78 in October and a minimum value of 0.22 in July. Also, Olmuş et al. (2022) studied the effect of incorporating photovoltaic-thermal solar collectors with rotary desiccant air conditioners integrated with heat recovery units and dew-point indirect evaporative coolers, and direct evaporative coolers. Guan et al. (2022) studied the influences of incorporating the solid rotary desiccant wheel with the liquid desiccant dehumidifier on the behavior of novel air-conditioners. Tian et al. (2022) presented a comprehensive review of the influences of incorporating rotary desiccant dehumidifiers with the heat pump for building air conditioning systems. Tsai and Wu (2022) theoretically evaluated the behavior of rotary desiccant wheels of air-conditioners in a humid environment. Wang et al. (2022) conducted a comparison study between the liquid desiccant air conditioner and the solid desiccant air conditioner under different climatic conditions in China. Singh and Das (2022) examined the influence of incorporating the solar energy-based desiccant with the variable refrigerant flow (VRF) air-conditioning system on the performance of VRF air condition systems. They conducted that solar energy-based desiccant-assisted VRF air condition systems could be saving power at a rate reached 23.9% compared to conventional VRF air condition systems. Chen and Shi (2022) studied the behavior of rotary desiccant air conditioners in the deep underground spaces. Liu et al. (2023) proposed the new desiccant-coated heat exchangers with higher efficiency for air-conditioners to improve their energy efficiency. Su et al. (2021) proposed a dehumidification system incorporated with the precooling and recirculating regenerative desiccant wheel suitable in hot and humidity regions. Su et al. (2022) theoretically studied the behavior of liquid desiccant air-conditioners. They found that the utilization of vacuum-assisted regeneration reduced the regeneration temperature and enable the utilization of low-grade energy or renewable.

Several studies are conducted, which primarily aim to develop desiccant air conditioning systems and improve their performance. Therefore, this research paper aims to provide a review of the latest technology in the design of desiccant air conditioners and their integration with other technologies, as well as the latest desiccant materials. Therefore, this study presents a review of desiccant air conditioners with a special focus on the design of desiccant air conditioners and desiccant materials, as well as the latest technology in integrating desiccant air conditioners with other hybrid energy technologies. This review is organized as follows: First, the overview is provided about the importance of using desiccant air conditioning systems compared to traditional vapor-compression refrigeration systems. In the second section, an overview is given of all desiccant materials used in desiccant air conditioners and the latest composite desiccant materials that have been reached. Section 3 provides a detailed review of the design of the desiccant air conditioner and the various circuits. Section 4 provides an overview of the latest hybrid technologies reached by recent studies, which were achieved by the integration of desiccant air conditioners with other hybrid energy technologies. Also, this section provides a detailed description of control improvement strategies utilized in hybrid desiccant air conditioners. Section 5 made recommendations for future work.

## Desiccant materials

**Fig. 1:**
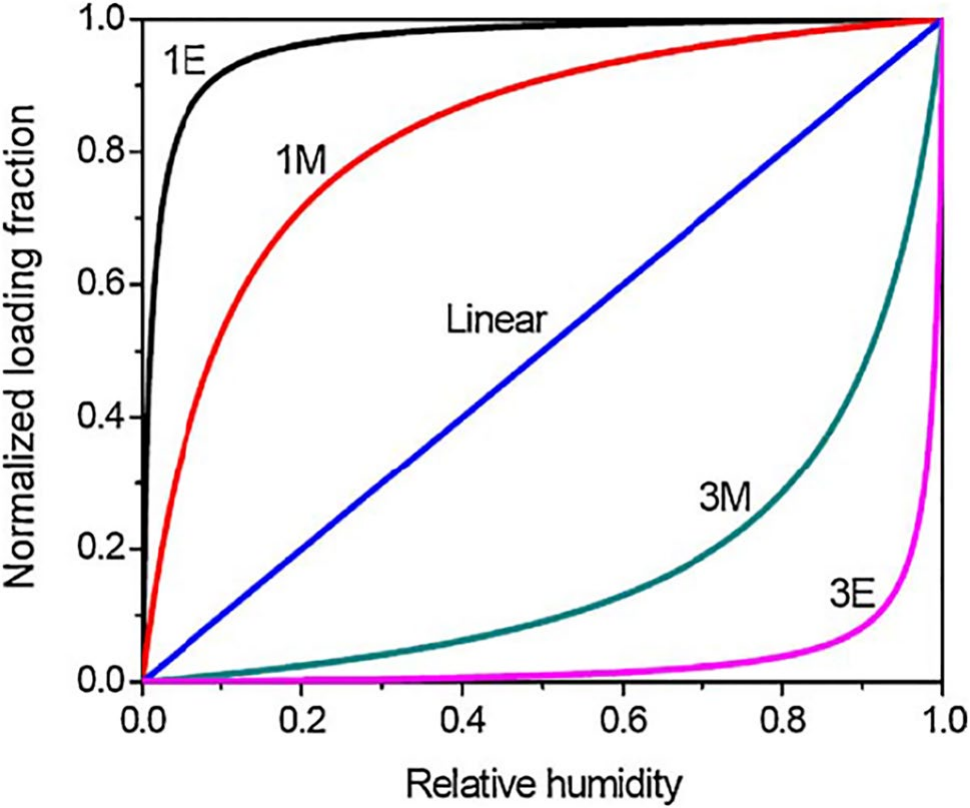
Comparison between the adsorption isotherms of type 1 moderate (1 M), type 1 extreme (1E), linear, type 3 extreme (3E), and type 3 moderate (3 M) (Collier et al. 1986; RK 1988)

Desiccant material is a hygroscopic material that attracts moisture due to a difference in vapor pressure. It can be classified into liquid or solid, synthetic or natural, rock or bio, chemisorption or physisorption, etc. The term chemisorption or physisorption refers to the bond strength between the adsorbent and adsorbate. Moisture removed from the air is usually considered to be decomposition adsorption due to the reduced strength of the bond between the absorbent and the adsorbent. The bond strength is preferred to be low in desiccant air conditioners for a perfect reactivation process. Due to the ability of desiccant material to adsorb water vapor, it is widely utilized in many applications such as hospital, pharmaceutical, industrial applications, drying food, and storage (Wurm et al. 2002). Desiccant materials are one of the main factors in the development of a desiccant air conditioner. The behavior of desiccant air conditioners is affected by the characteristics of the desiccant material being utilized (Collier 1989), there are three main factors for selecting a suitable desiccant material:

1. The ability of desiccant materials to adsorb water vapor.
2. The desiccant material should be reactivated at low temperatures.
3. Desiccant materials play a crucial role in developing desiccant air conditioning systems. The characteristics of the selected desiccant material have an impact on the desiccant air conditioner’s performance, where the desiccant materials include activated alumina, activated carbon, silica gel, molecular sieve, calcium chloride, lithium chloride, etc. (Waugaman et al. 1993). The desiccant materials type 1 M material represents the ideal desiccant material for air conditioning applications, as presented in Fig. 1. As shown in Fig. 1, the normalized loading fraction of type 1E material is higher than that of type 1 M material, but the type 1E material is more difficult for reactivation due to the nearly complete loading at lower relative humidity RH (Collier et al. 1986; RK 1988), where the normalized loading fraction is defined as the ratio of actual water content in desiccant at corresponding RH to maximum water content in desiccant at 100% RH.

Recent studies in solid desiccant materials consist of four aspects:

1. Modified conventional desiccant materials (Ding et al. 1998; Chung and Chung 1998; Knez and Novak 2001; Yano and Fukushima 2003; Fang et al. 2004; Pramuang and Exell 2007; Abd-Elrahman et al. 2011; Hamed et al. 2013).
2. Natural rock-based desiccant materials (White and Bussey 1997; Montes-H and Geraud 2004).
3. Bio-desiccant materials (Ladisch 1997; Beery and Ladisch 2001; Zhou et al. 2003; Khedari et al. 2003).
4. Composite desiccant materials (Kuma and Okano 1989; Aristov et al. 1996b, a; Tokarev and Aristov 1997; Gordeeva 1998; Gordeeva et al. 1999; Thoruwa et al. 2000; González et al. 2001; Tokarev et al. 2002b, a; Liu et al. 2003; Zhang 2003; Liu and Wang 2003; Jia et al. 2006, 2007; Aristov 2007).

### Common sorbents and their properties

#### Silica gel

Silica gel is one of the commonly widely utilized sorbent materials due to its ability to absorb water and be reactivated at relatively low temperatures (Knez and Novak 2001; Chung and Chung 1998; Pramuang and Exell 2007). The chemical composition of silica gel is silicon dioxide (SiO_2_.xH_2_O). Silica gel is available in different pore sizes and the surface area per unit mass decreases as pore size increases. Where a surface area reached 650 m^2^/g at pore size 2-3 nm (type A), the type B (0.7 nm pore size) was used for relative humidity greater than 50% and adsorption heat 2800 kJ/kg (Srivastava and Eames 1998; Sultan et al. 2015).

#### Activated alumina

Activated alumina is formed by dehydroxylating aluminum hydroxide and its surface area ranges between 150 and 500 m^2^/g at pore sizes 1.5–6 nm and adsorption heat of 3000 kJ/kg (Srivastava and Eames 1998; Sultan et al. 2015). Many researchers studied the characteristics of this sorbent material under different inlet conditions and different system configurations. Abd-Elrahman et al. (2011) experimentally conducted a behavior of radial flow desiccant bed with activated alumina. In this study, 39.860 kg of activated alumina as spherical particles with a 4 mm average diameter distributed in 90 cm length of a hollow cylindrical bed with inner/outer diameters of 10.8/27.8 cm, respectively, they found that air with moisture content ranging from 18.7 to 12.5 g/kg could be dehumidified to 1.2 g/kg using activated alumina. Hamed et al. (2013) theoretically and empirically investigated the behavior of activated alumina in radial flow desiccant bed, they found an acceptable agreement between the experimental and theoretical results.

#### Activated carbons and bio desiccants

A lot of absorbent materials are produced from biomass with a good ability to adsorb water vapor (Beery and Ladisch 2001; Ladisch 1997; Zhou et al. 2003; Khedari et al. 2003). Khedari et al. (2003) experimentally conducted a behavior of utilizing coconut coir and durian peel as a desiccant material, the results showed that under air conditions (32 °C and 75% RH), durian peel and dry coconut coir can absorb 17 and 30 g H_2_O per 100 g dry product, respectively. A comparison between silica gel and dry coconut coir presented that the adsorption rate of silica gel is higher than coconut coir by 5 g/h per 100 g dry product at 60 min operating time and airflow rate of 84 m^3^/h. Also, biomass used to produce activated carbon from different sources such as coconut shells, walnut shells, almond shells, pistachio shells, oil palm shells, cherry stones, coffee endocarp, and chickpea husk ( Olivares-Marín et al. 2006; Hayashi et al. 2002b, a; Nabais et al. 2008). Shimooka et al. (2006, 2007) experimentally improved activated carbon adsorption capacity using some treatments and the results confirmed that oxidized activated carbon had an adsorption capacity between 1.2 and 1.9 times silica gel.

#### Liquid and polymer desiccants

Liquid desiccants such as calcium chloride (CaCl_2_), lithium chloride (LiCl), lithium bromide (LiBr), and triethylene glycol are used in different desiccant air conditioning systems due to their ability to reactivate at low temperatures from 60 to 75 °C, these provide the opportunity to benefit from low-grade waste heat and solar energy ( Liu et al. 2007, 2011; Fumo and Goswami 2002; Patnaik et al. 1990; Mei and Dai 2008; Koronaki et al. 2013). For polymer desiccants, a new polymeric desiccant called super desiccant polymer is presented by Lee and Lee (2012), they experimentally studied its characteristics and found that the capacity of polymer sorption is larger than various types of silica gel by 2 to 3 times. White et al. (2011) empirically compared the behavior of a conventional silica gel desiccant wheel with a desiccant wheel containing (zeolite and super adsorbent polymer), they conducted that utilization of super absorbent polymer achieved greater dehumidification than silica gel at a low reactivation temperature of 50 °C. Mathiowitz et al. (2001) produced a new concept of desiccant preparation that can be used in pharmaceutical and industrial applications.

#### Molecular sieve

Molecular sieves or synthetic zeolite is an aluminosilicate mineral which used in many air conditioning applications as a desiccant material due to its ability to adsorb water vapor (White and Bussey 1997; Kodama 2010; La et al. 2010; Kubota et al. 2011, 2013; Rao et al. 2012; Karamanis and Vardoulakis 2012). White and Bussey (1997) experimentally studied the adsorption characteristics of a clinoptilolite which is a natural zeolite and the results showed that the zeolite uptake of water is less than that of silica gel. To improve the desiccant material’s characteristics such as the ability of the material to adsorb water and reactivated at low temperatures, silica gel and zeolite are used to develop a composite desiccant material (La et al. 2010).

#### Composite materials

**Fig. 2:**
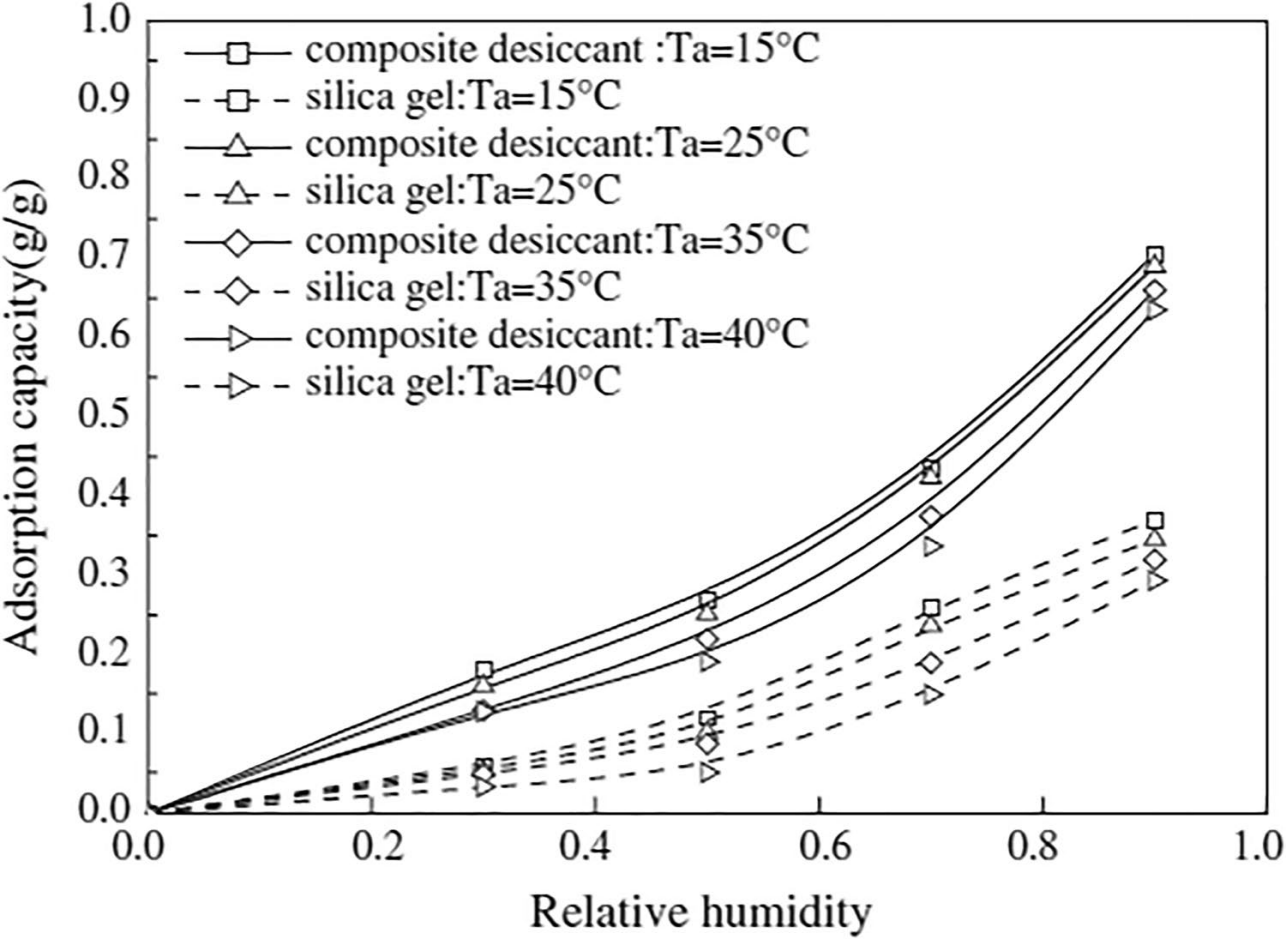
The equilibrium adsorption curves (Jia et al. 2007)

Many efforts have been done on composite desiccants because of their characteristics which approach the type 1M material that has the perfect adsorption capacity and is regenerated at low temperatures. Jia et al. (2006, 2007) empirically investigated the comparison between two wheels, one with silica gel and the other with lithium chloride (LiCl) and silica gel, the outcomes showed that an adsorption capacity of composite materials is one to two times greater than silica gel as shown in Fig. 2 and for the same moisture, the second wheel being reactivated at a lower temperature.

**Fig. 3:**
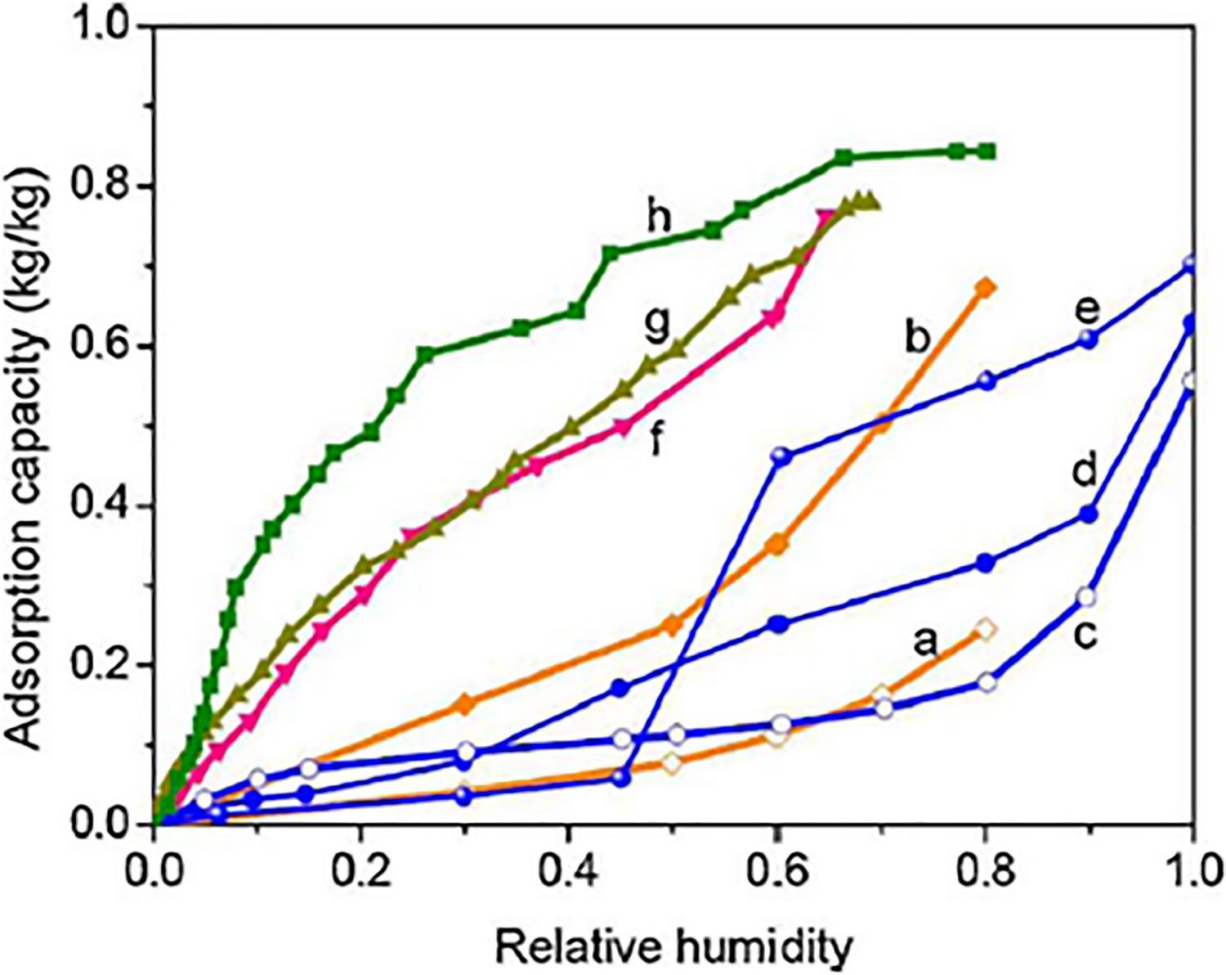
Isothermal water vapor absorption for **a** silica gel, **b** LiCl-silica gel, **c** sepiolite, **d** carbon-sepiolite (activation by steam), **e** carbon-sepiolite (activation by KOH), **f** CaCl_2_–SiO_2_ sol–gel, **g** CaCl_2_–MCM-41, and **h** LiBr-silica gel (La et al. 2010)

Also, some composite materials have been produced by Aristov et al. (1996a, b), Tokarev and Aristov (1997), Gordeeva (1998), Gordeeva et al. (1999), and Tokarev et al. (2002b) using silica gel and some hygroscopic salt such as calcium chloride (CaCl_2_) and lithium bromide (LiBr). The results showed that about 80% of water adsorbed by the developed composite materials could be desorbed with a regeneration temperature between 80 and 90 °C. Liu et al. (2007, 2011) empirically studied the behavior of a new composite sorbent SiO_2_.xH_2_O.yCaCl_2_ that was a combination between calcium chloride (CaCl_2_) and silica gel and compared the results with other common desiccant materials. They found that the reactivation temperature of composite desiccant was about 60–80 °C and the adsorption capacity reached 0.4 g H_2_O/g dry adsorbent at ambient conditions (40% RH and 25 °C) and, which reached 2.1 times of microporous silica gel, 5.7 times of microporous silica gel, 6.8 times of activated carbon and 1.9 times of synthetic zeolite 13X. Synthesized zeolite and silica gel-based composite desiccant presented by Kuma and Okano (1989). This composite material combined the advantages of zeolite and silica gel; the results showed that a composite desiccant material exhibited an excellent ability to adsorb water vapor and thus, this material could be applied to dehumidify the air. Moreover, two types of sepiolite-based composite desiccant materials, sepiolite-calcium chloride, and sepiolite-activated carbon have been prepared by González et al. (2001). The first increases the ability of sepiolite to moisture control and improved the absorption capacity. The second is an increase in the relative humidity range in applications of sepiolite as a humidity controller between 89 and 39% (Thoruwa et al. 2000), and the results of the regeneration characteristics and moisture sorption showed that this material can be utilized in solar crop drying. Tokarev et al. (2002a) introduced a new composite desiccant by combining calcium chloride (CaCl_2_) with a mesoporous host matrix MCM-41, this allowed two kinds of sorption behavior (solid adsorption and liquid absorption) allowing combining their advantages. The results showed that the composite sorbent could absorb 0.75 g H_2_O/g dry sorbent which ensures the high values of energy capacity (2.1 kJ/g) and it was found that at regeneration temperature between 70 and 120 °C, most moisture content can be removed from the composite desiccant (Fig. 3) (La et al. 2010). It showed that a composite sorbent greatly improves an adsorption capacity.

### Progress in solid desiccant air conditioning

As sorbents can be either liquid or solid, desiccant air conditioners are classified into two categories, namely, solid desiccant air conditioners and liquid desiccant air conditioners. Being beneficial in handling the latent load, using low-grade energy, and being CFC-free, these technologies have been widely used. Especially, rotary desiccant air conditioners work continuously and have less chance of corrosion. So far, many mathematical simulation studies of rotary desiccant air conditioners have been conducted (Zhang et al. 1996; Nia et al. 2006; Ge et al. 2008b), empirically conducted (Ge et al. 2008a, 2009; Kabeel 2007), thermodynamic analysis (Kanoǧlu et al. 2004, 2007; Shen and Worek 1996), and practical application ( Casas and Schmitz 2005; Henning et al. 2001; Sand and Fischer 2005). Now, for the development of rotary desiccant air conditioning technology, researchers work on two axes: (1) advanced desiccant materials as illustrated in section 2; (2) optimum system configurations to decrease energy consumption by using renewable energy sources and then increasing system performance.

#### Principle of rotary desiccant dehumidifier

**Fig. 4:**
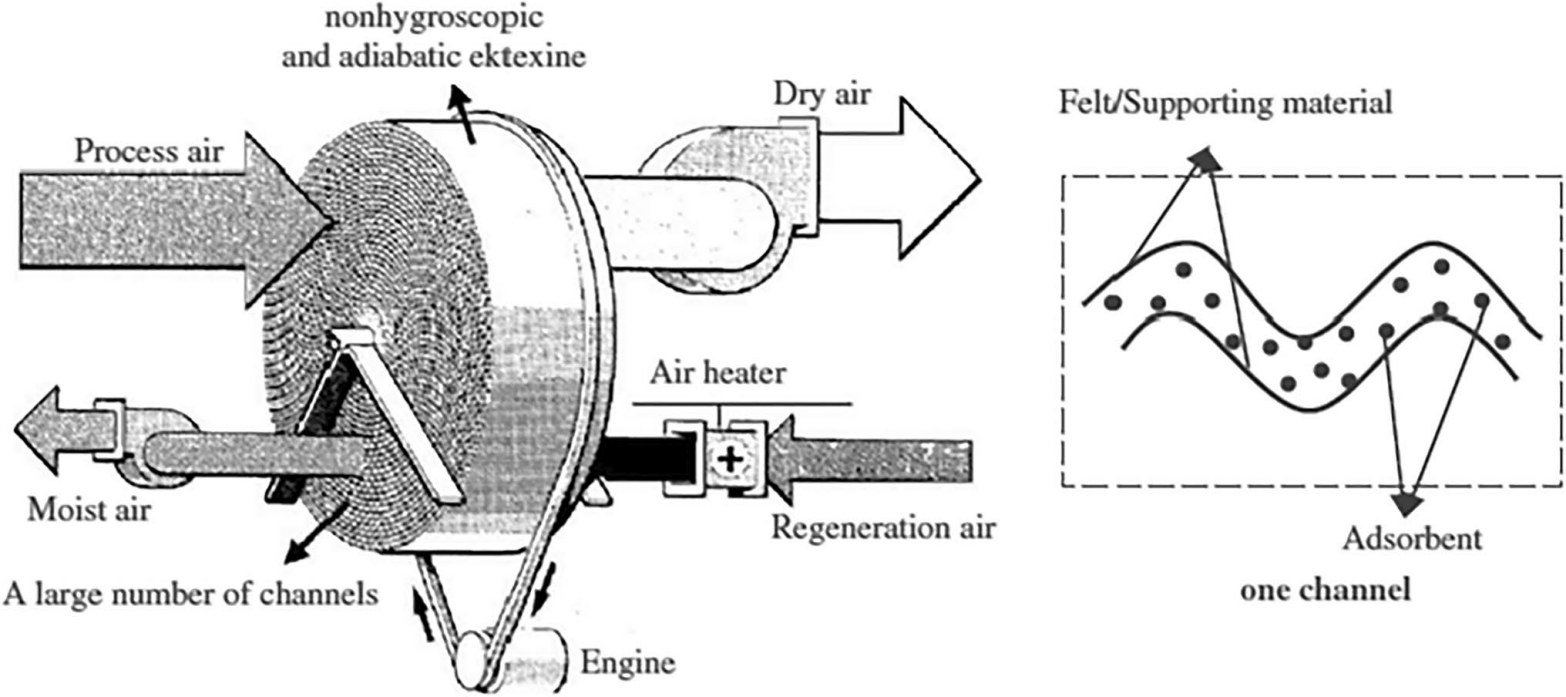
Schematic diagram of the rotary desiccant dehumidifier (Ge et al. 2008b)

In a rotary desiccant wheel, mass and heat transfer takes place between moist air and sorbent material at a low rotation speed (8–10 revolutions per hour). The rotary wheel is made from a honeycomb with a thin layer of sorbents, and it is divided into two sections, one for reactivation and the other for a process (Yadav and Bajpai 2011). The operation of the rotary wheel is summarized as presented in Fig. 4 (Ge et al. 2008b): as the outdoor air flows through a process section, the moisture is transported from air to sorbents which are distributed in the flow channels, this transfer is to the vapor pressure difference between the sorbents and air streams. During this process, due to the adsorption of latent heat, the sorbent’s temperature increases, as well as the heat will be transferred by convection to the airstream which will increase the outlet airstream temperature. Reactivation process, when absorbent particles get saturated with water, they need to be reactivated. This is done by using a heat source (electric heater or solar/wasted heat) to heat the sorbents by passing the reactivation air through it; the temperature of reactivation air depends on the type of sorbent used. In the adsorption process, the latent heat is converted to sensible heat and does not produce useful cooling. So, to obtain a cooling effect, the rotating wheel is combined with the auxiliary coolant (evaporative cooler) to remove sensible heat. As illustrated, the system configuration affects on the desiccant air conditioner’s performance, and different types of rotary desiccant air conditioners are introduced in the next section.

#### Solid desiccant cooling cycles

##### Pennington cycle

**Fig. 5:**
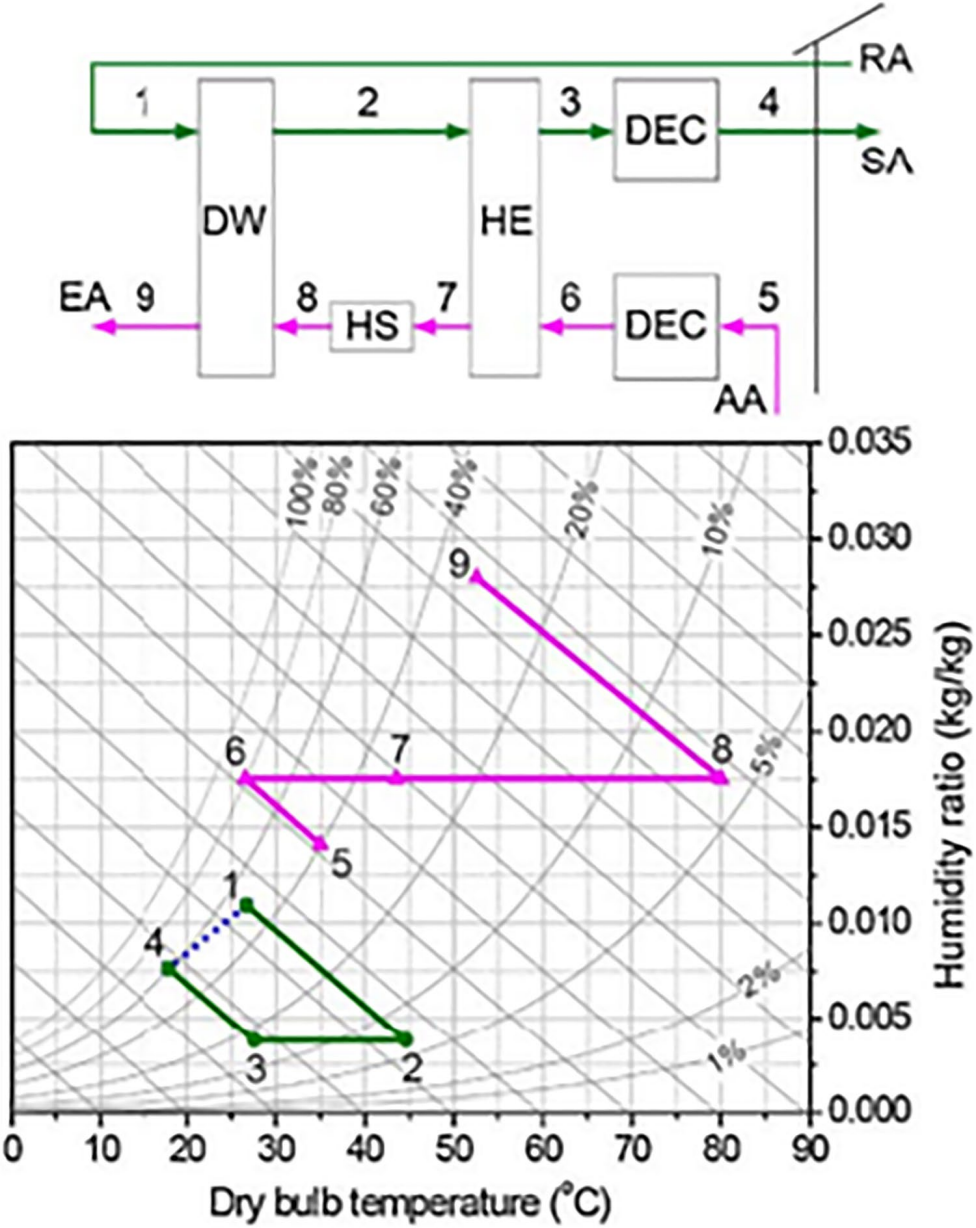
Pennington cycle

Pennington (1955) introduced the first desiccant air conditioner cycle which is called the Pennington cycle or ventilation cycle. As illustrated in Fig. 5, outdoor air at point 1 passes to the rotary wheel, as a result, it is dehumidified and its temperature increases due to adsorption heat impact. Through the process from state points 2 to 3, the hot dry air is sensibly cooled in the heat exchanger. Then, the process air flows to the evaporative cooler where it is evaporatively cooled, humidified, and supplied to the room at state 4. On the reactivation air side, return air at point 5 passes to another evaporative cooler where it is cooled and humidified. Through the process from state points 6 to 7, the humidified cooled air will exchange the heat with process air in the heat exchanger, as a result, it is preheated and process air is precooled. Then, warm air is heated from points 7 to 8 by the heat source and passes to a wheel to reactivate the sorbent material, as a result, it is humidified, and its temperature is decreased and exhausted at state point 9.

##### Modified ventilation cycle

**Fig. 6:**
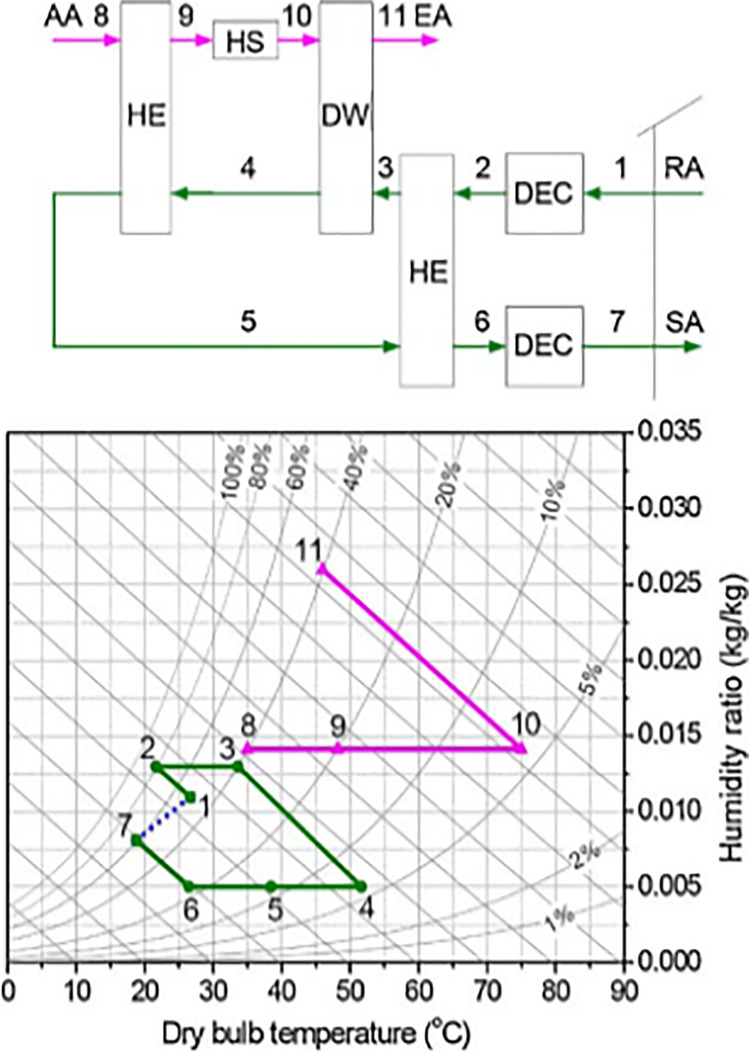
Ventilation cycle

The modified ventilation cycle presented in Fig. 6 is introduced as the building exhaust of room air in the previous cycle. The difference between the Pennington cycle and the modified ventilation cycle is using the ambient air for regeneration in the modified ventilation cycle instead of return air and this decreases the thermal performance due to the temperature and humidity of outdoor air is usually greater than return air.

##### Recirculation and Dunkle cycles

**Fig. 7:**
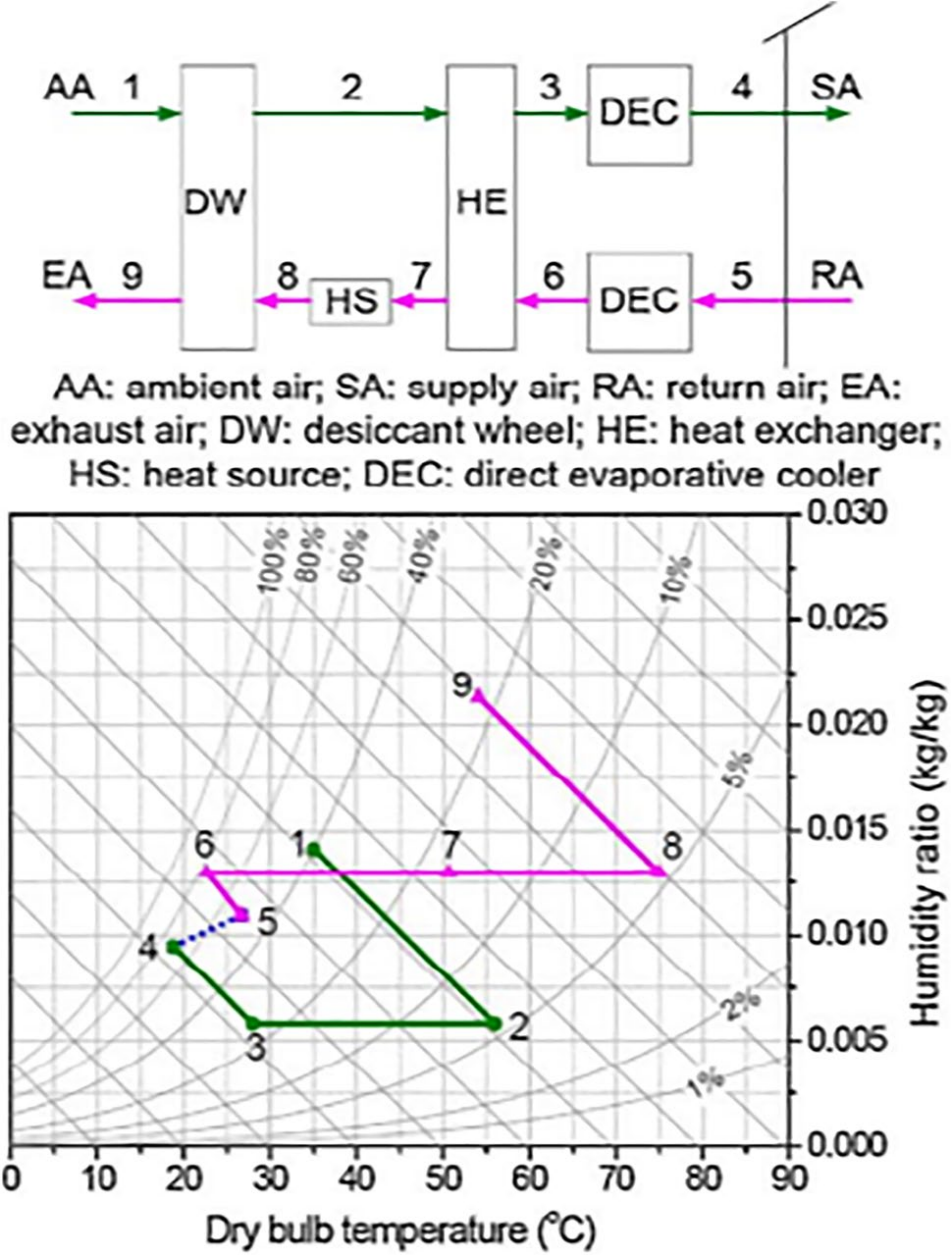
Recirculation cycle

To increase cooling capacity and COP, the recirculation cycle reuses the return air as the process air as shown in Fig. 7. Outdoor air is still utilized for reactivation and then exhausted to the atmosphere. The coefficient of performance for this cycle is 0.8 or less (Waugaman et al. 1993).

**Fig. 8:**
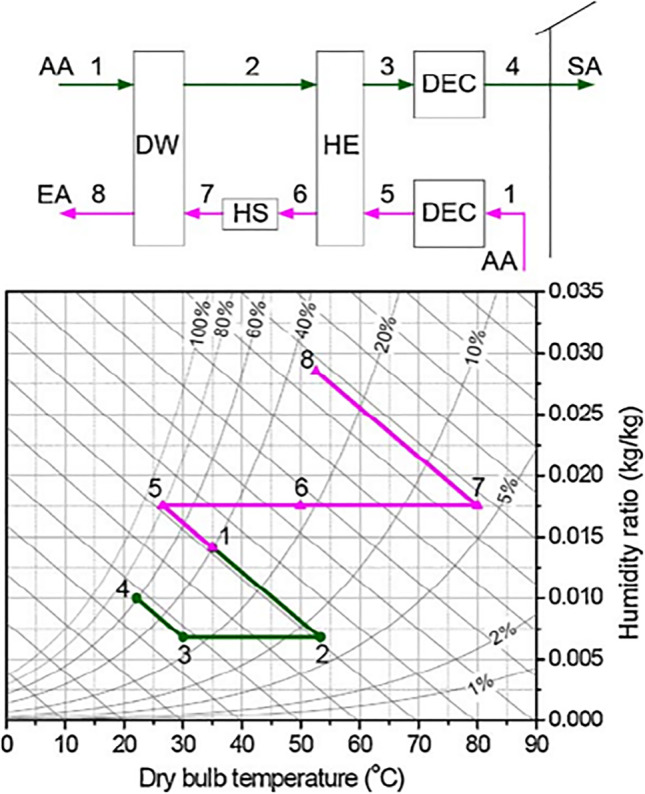
Dunkle cycle

In another way to increase the cooling capacity, Dunkle (1965) added a heat exchanger in the recirculation cycle that can provide a colder process air to the main heat exchanger as presented in Fig. 8. Like the recirculation cycle, the Dunkle cycle also reuses return air as a process air and uses outdoor air in reactivation process. The major drawbacks of recirculation and Dunkle cycles are lack of fresh air. It should be noted that fresh air represents an additional cooling load not only means comfort and most applications do not require to be supplied with total fresh air, so fresh air must be maintained at the required level for better indoor air quality and good system performance.

##### SENS, REVERS, and DINC cycles

**Fig. 9:**
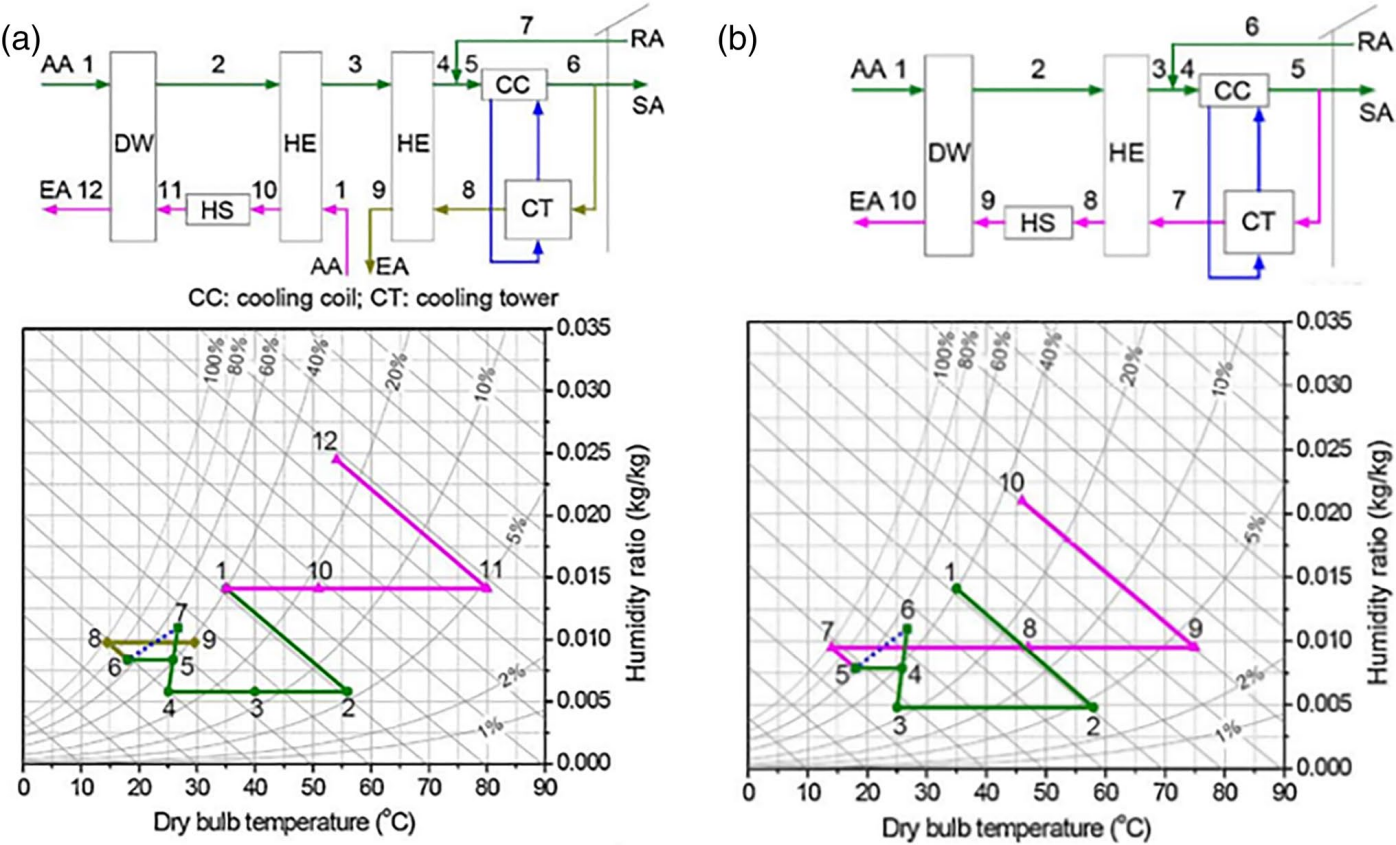
**a** SENS cycle and **b** REVERS cycle

To overcome lacking fresh air in previous cycles, Maclaine-cross (1974) introduced an advanced simplified desiccant cycle called SENS cycle. Figure 9a shows the outdoor air first dehumidified in a wheel and its temperature increase due to adsorption heat impact. Then, the hot dry air gets cooled in two heat exchangers connected in tandem. Afterward, the process air is mixed with the return air and cooled after that in a heat exchanger using cold water. The mixed cooled air is divided into two parts. The first part flows to a conditioned space and the other part is used to cool water in the cooling tower. On the reactivation side, outdoor air is pre-heated in the heat exchanger, then drawn to a wheel and exhausted back outdoors. The coefficient of performance of SENS cycle is above 2.0, under weather conditions (26% RH and 26 °C). Produced a coefficient of performance of about 2.45 (Waugaman et al. 1993).

**Fig. 10:**
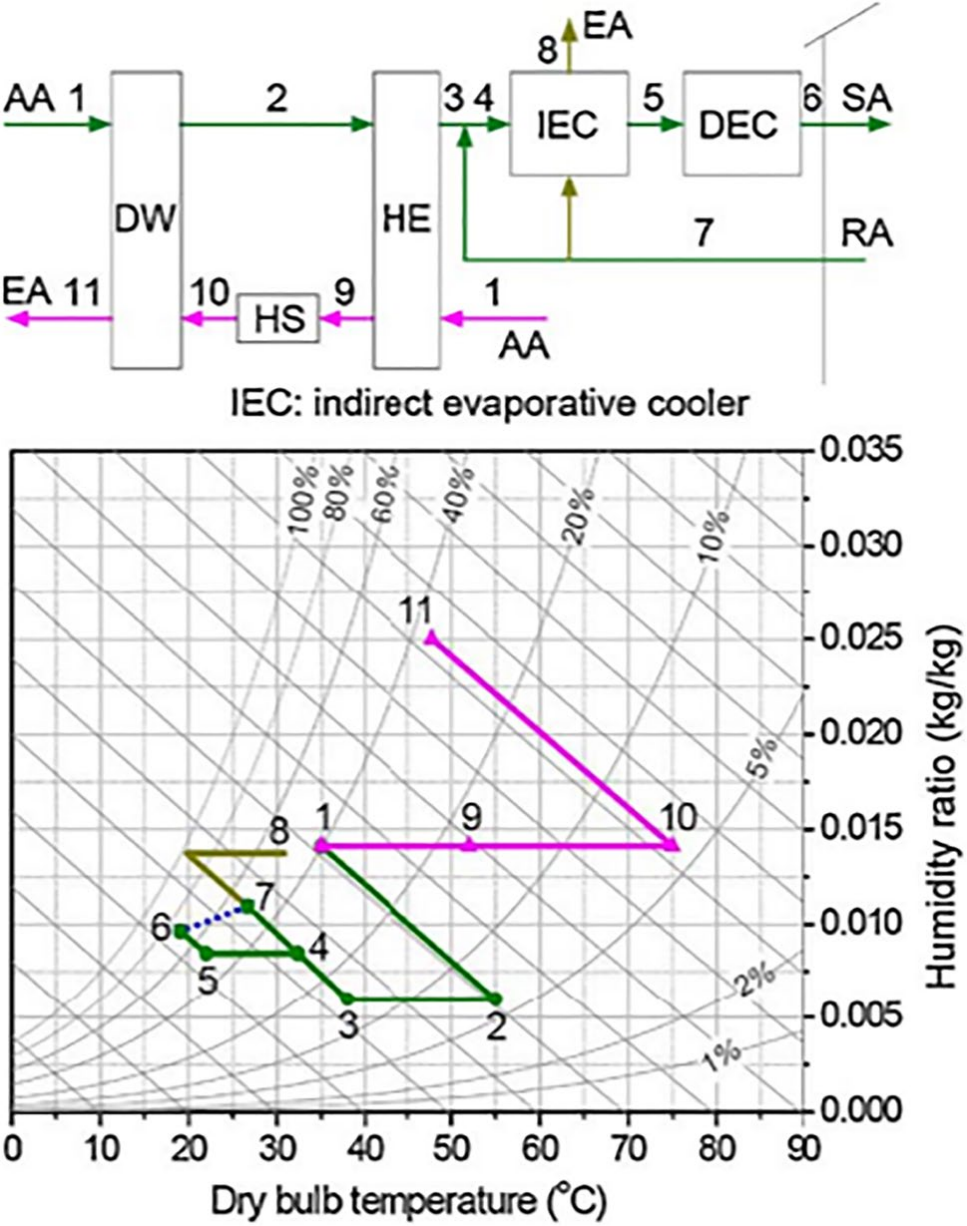
DINC cycle

To simplify SENS cycle, the heat exchanger is removed and then the REVERS cycle (Maclaine-Cross 1985) is introduced as shown in Fig. 9b. For more simplification, the cooling tower in the REVERS cycle is replaced by an indirect-direct evaporative cooler (DINC cycle) (Waugaman and Kettleborough 1987) as shown in Fig. 10. The difference between the DINC cycle and modified ventilation cycle is the indirect evaporative cooler that exists in DINC cycle. The coefficient of performance of the DINC cycle is above 1.6 under ARI conditions (Waugaman and Kettleborough 1987).

##### Multi-stage cycles

**Fig. 11:**
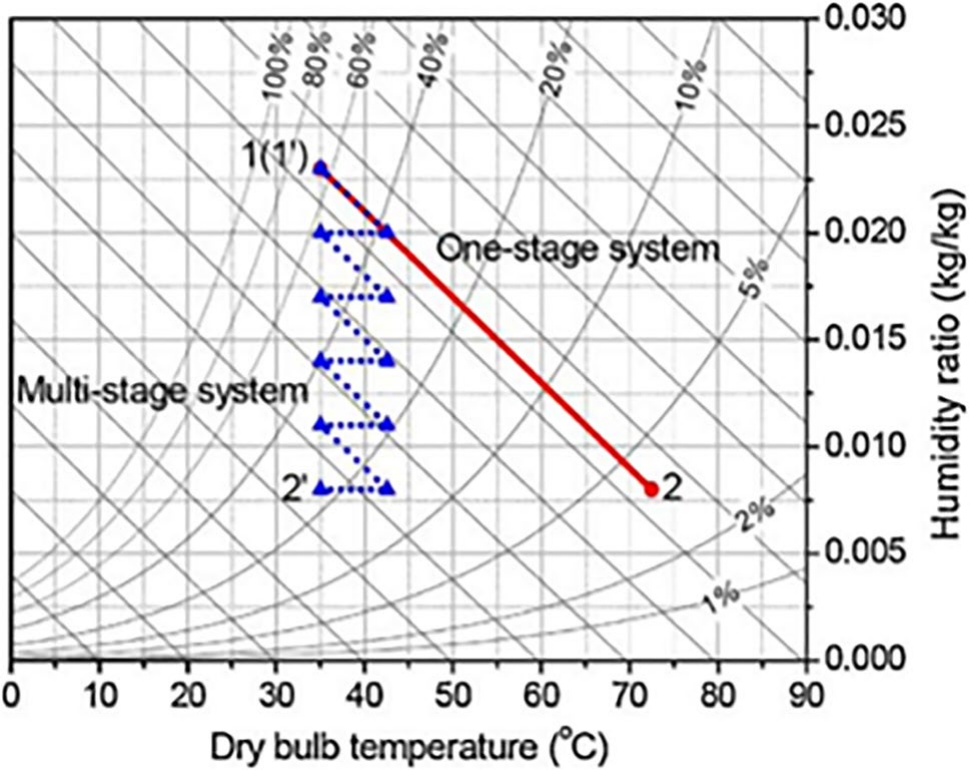
Psychometric chart of a multi-stage and one-stage system

Multi-stage cycles are proposed to compact the adsorption heat effect that increases the process air temperature during the dehumidification process, this is done based on isothermal dehumidification which decreases the process air temperature, increasing adsorption capacity, and lowers regeneration temperature. The thermodynamics procedure of the air would be close to isothermal when it flows alternatively over desiccant wheels and intercoolers. Figure 11 illustrates psychometrically the difference between an ideal multi-stage system and a one-stage system.

**Fig. 12:**
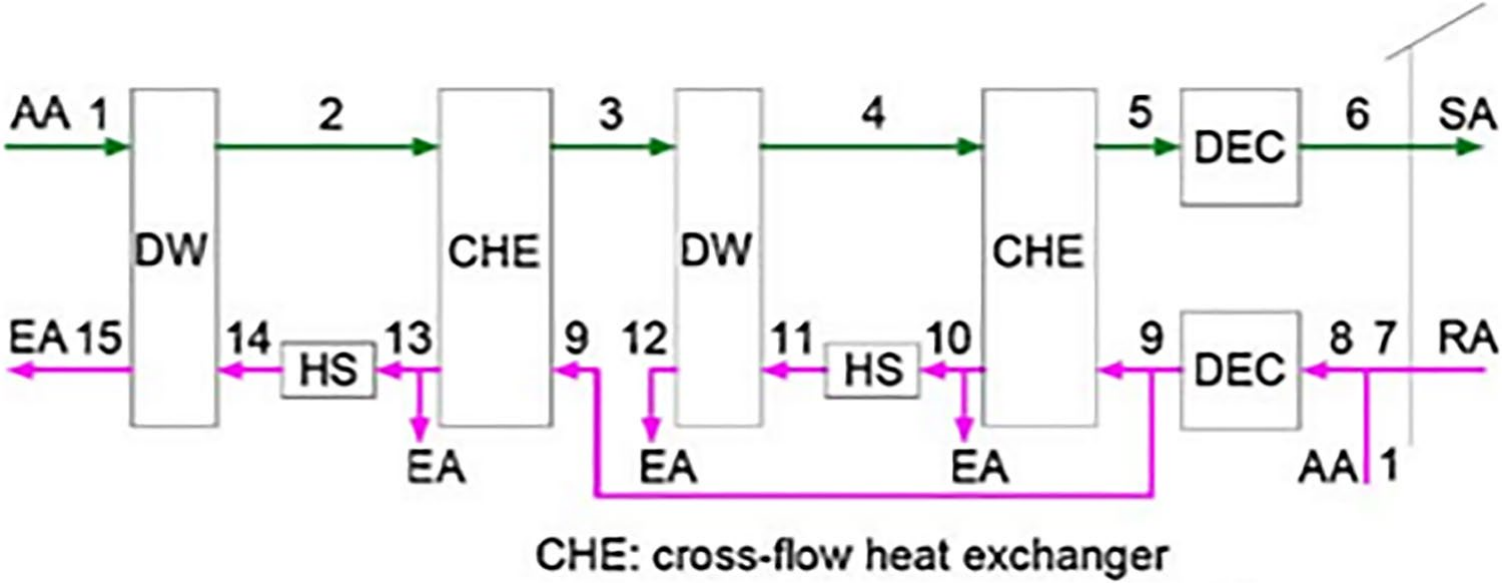
Two-stage desiccant system

**Fig. 13:**
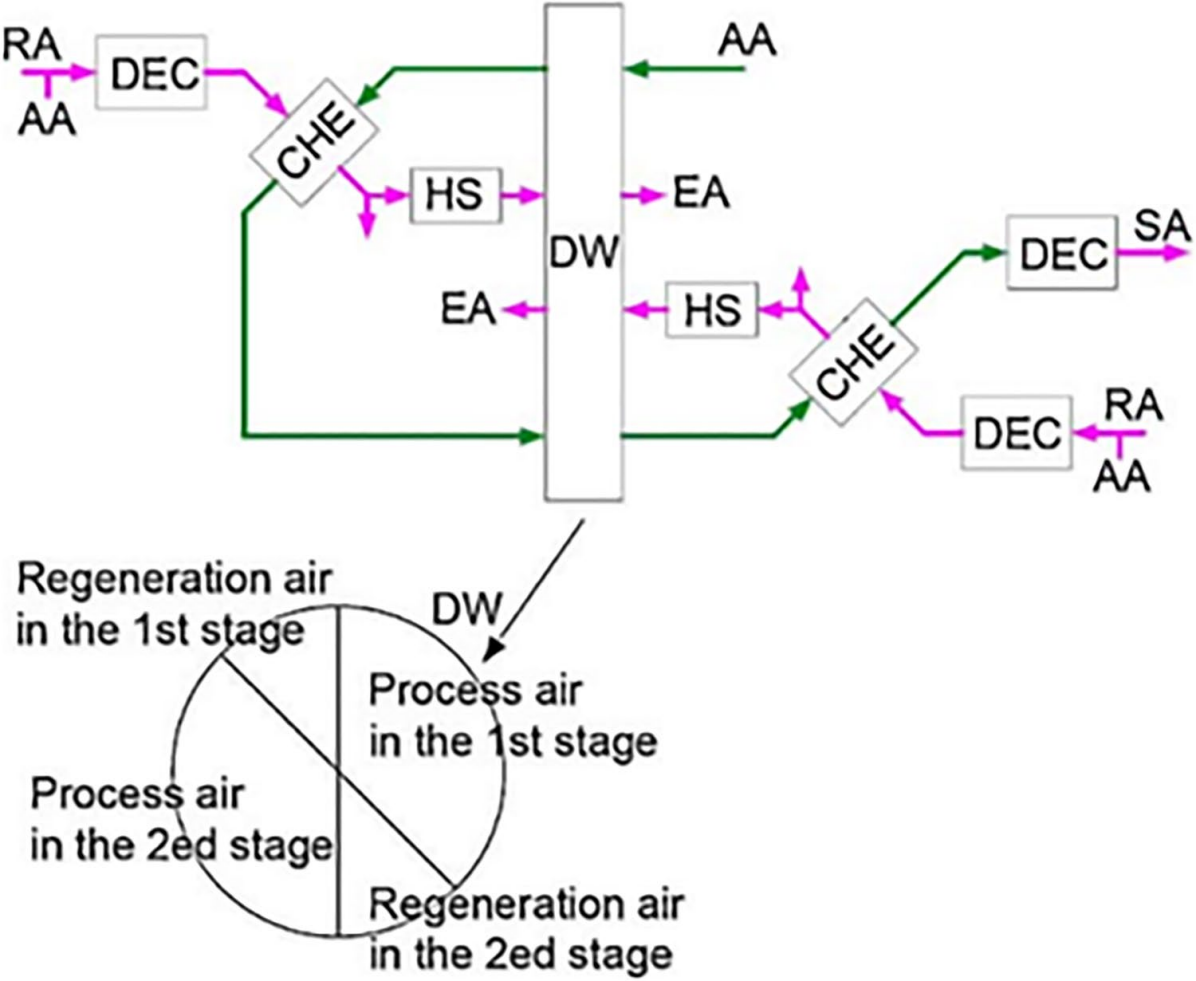
One rotor, two-stage desiccant wheel

**Fig. 14:**
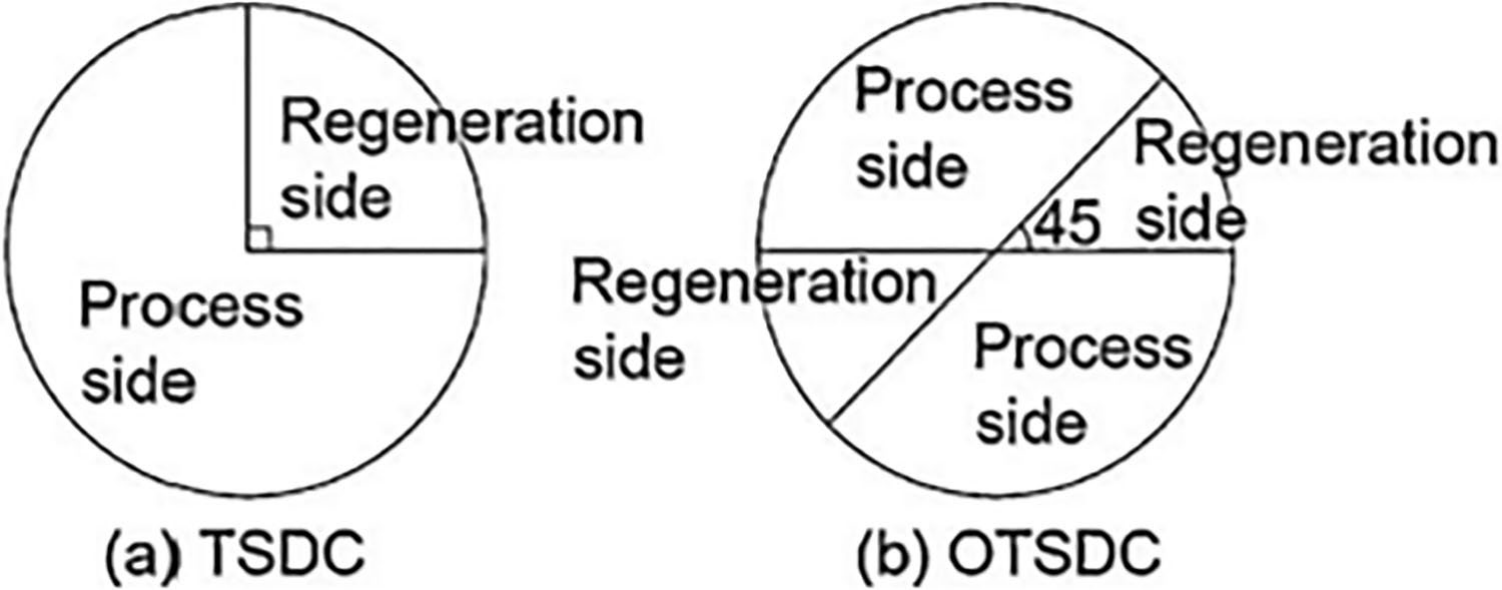
Schematic of **a** TSDC and **b** OTSDC [13]

**Fig. 15:**
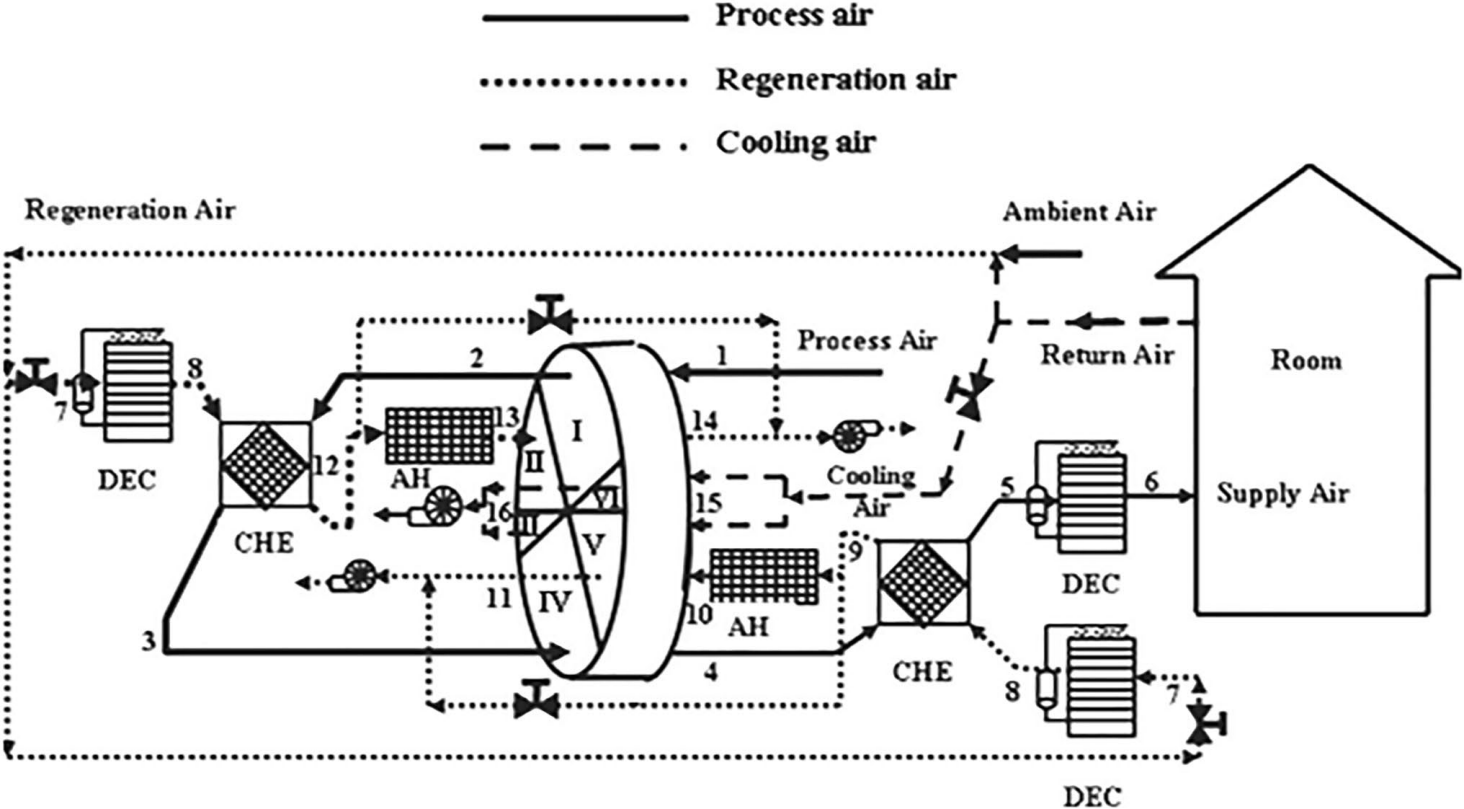
Schematic of solar hybrid air conditioner using one-rotor six-stage wheel (Elzahzby et al. 2014a)

**Fig. 16:**
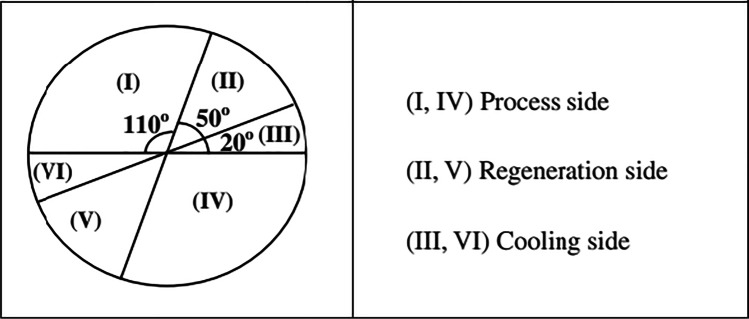
Cross-section area of six stage rotor wheel (Elzahzby et al. 2014b)

Two types of two-stage systems are introduced by Ge et al. (2008a, 2009), one is two-stage desiccant cooling (TSDC) based on two rotary wheels and the other is one-rotor two-stage desiccant cooling (OTSDC) using one rotary wheel, as shown within Figs. 12 and 13. The division of the cross-section of a wheel is the main difference between the two cycles. As seen in Fig. 14, the desiccant wheel of TSDC is divided into two parts: one for reactivation and the other for the process like a conventional desiccant wheel while the OTSDC wheel is divided into four parts: two for reactivation and two for the process. Composite materials (lithium chloride-silica gel) have been utilized in two systems and incorporated the internal coolers to increase system performance. The results confirmed that the two systems can operate with heat above 50 °C and get a coefficient of performance over 1.0. For the TSDC system, a required regeneration temperature decreased from 100 to 70 °C to reach moisture removed about 6 g/kg compared with the traditional one-stage system under summer conditions. Note that, the size of the OTSDC system is half of the TSDC system which makes it suitable for residential buildings. As continue in multi-stage cycles, Elzahzby et al. (2014b, a) introduced a one-rotor six-stage system whose wheel consists of two-stage for pre-cooling, two-stage for dehumidification, and two-stage for reactivation as shown in Figs. 15 and 16. The results showed that the mathematical model was validated with empirical data in (Ge et al. 2008a) and the operating parameters of this study are (wheel divided into six stages as illustrated above, regeneration velocity 2.5 m/s and temperature 90 °C).

### Hybrid solid desiccant air conditioners

**Fig. 17:**
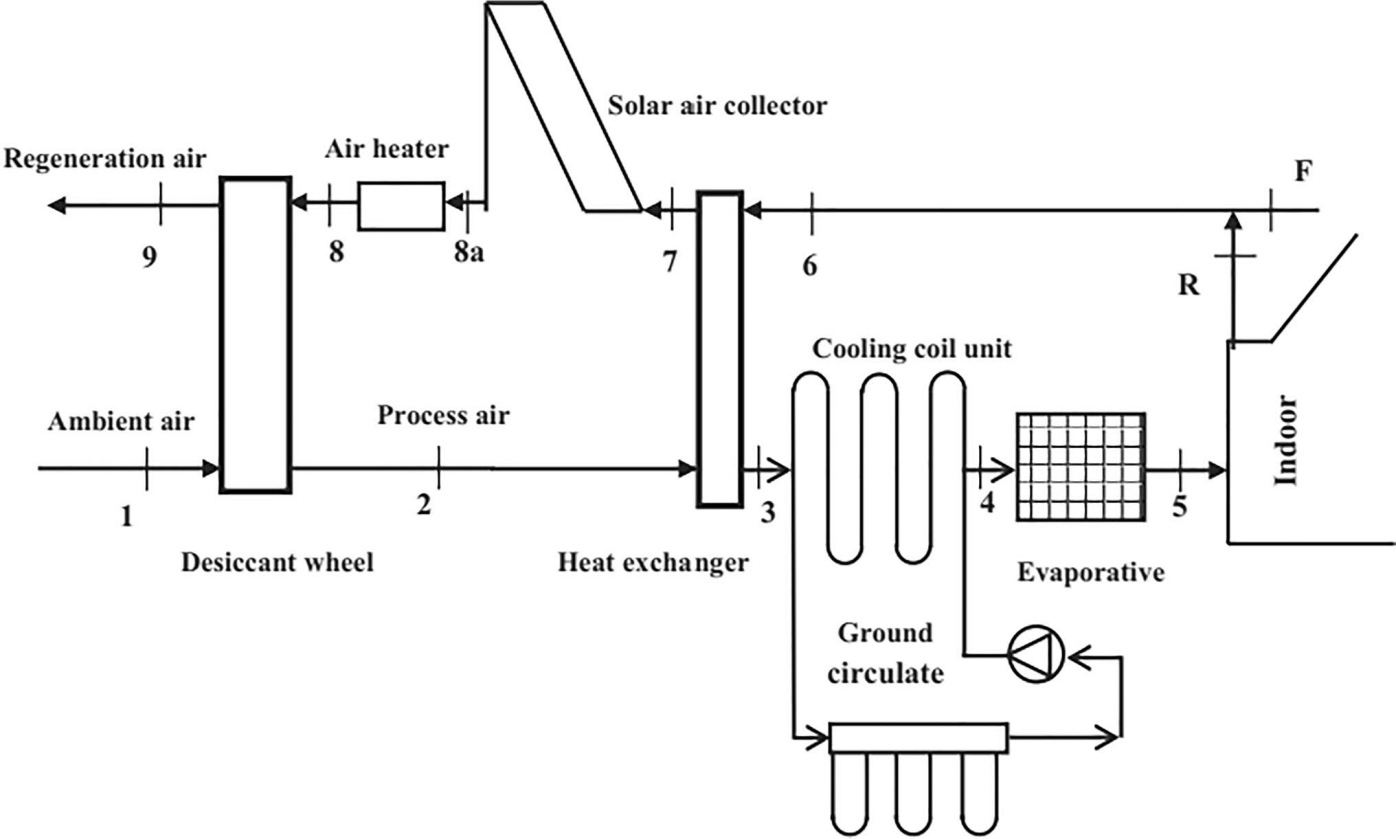
Solar desiccant air conditioner coupled with air solar collectors and a source of geothermal energy (El-Agouz and Kabeel 2014)

**Fig. 18:**
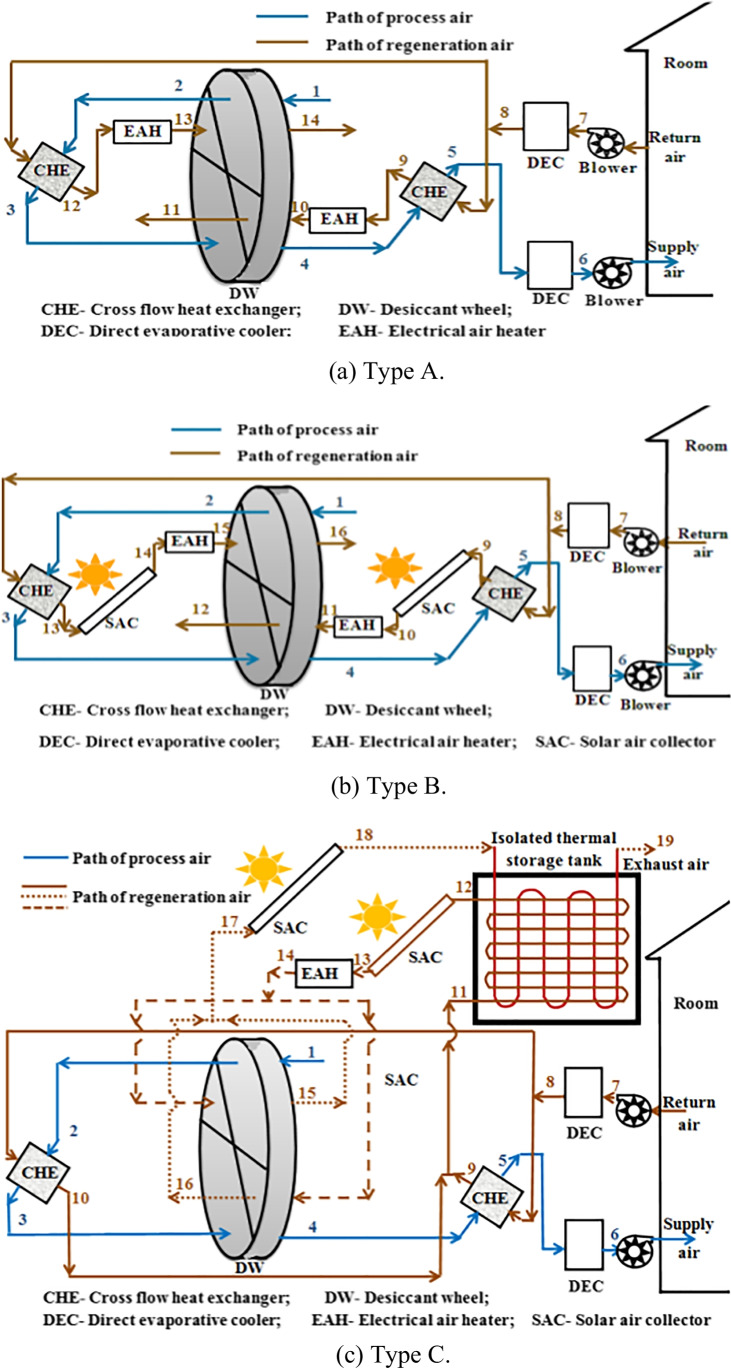
Solar assisted desiccant air conditioner: **a** type A, **b** type B, and **c** type C (Kabeel and Abdelgaied 2018)

**Fig. 19:**
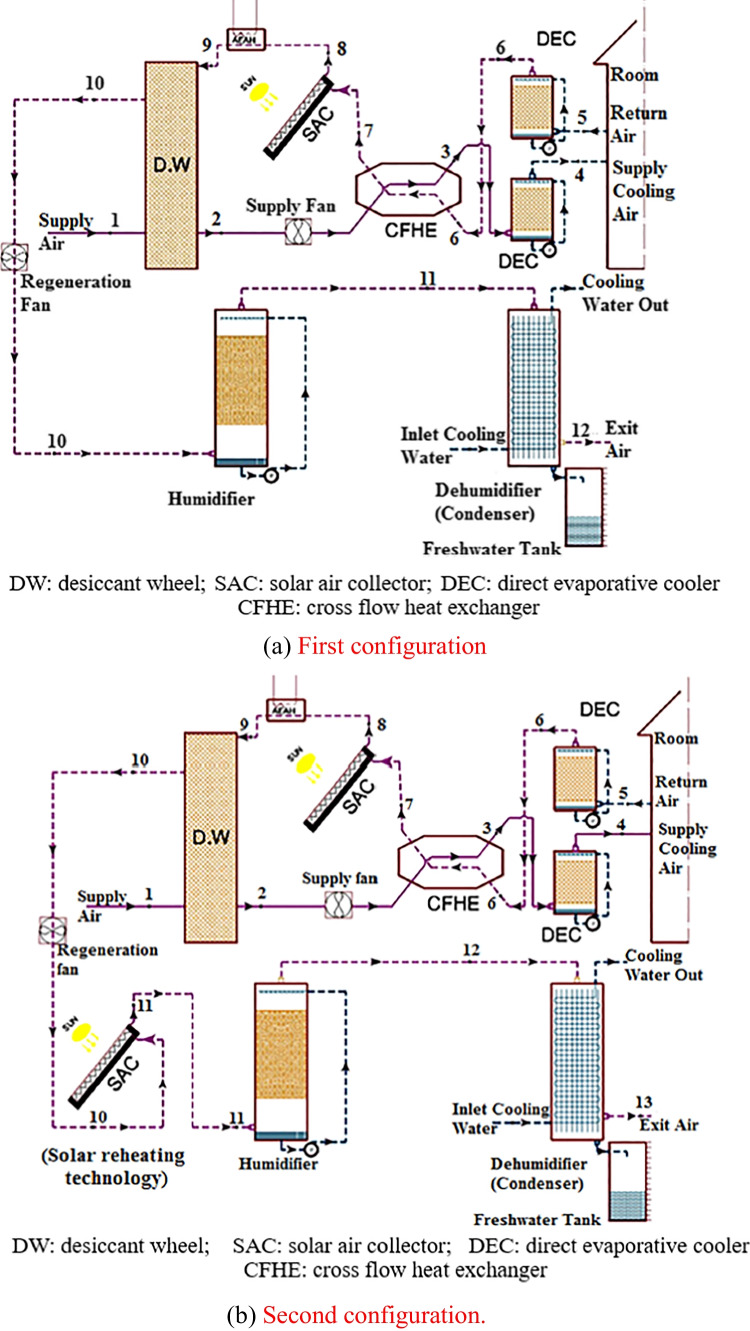
Hybrid solar desiccant air conditioner incorporated with HDH desalination unit: **a** first configuration and **b** second configuration (Abdelgaied et al. 2019)

**Fig. 20:**
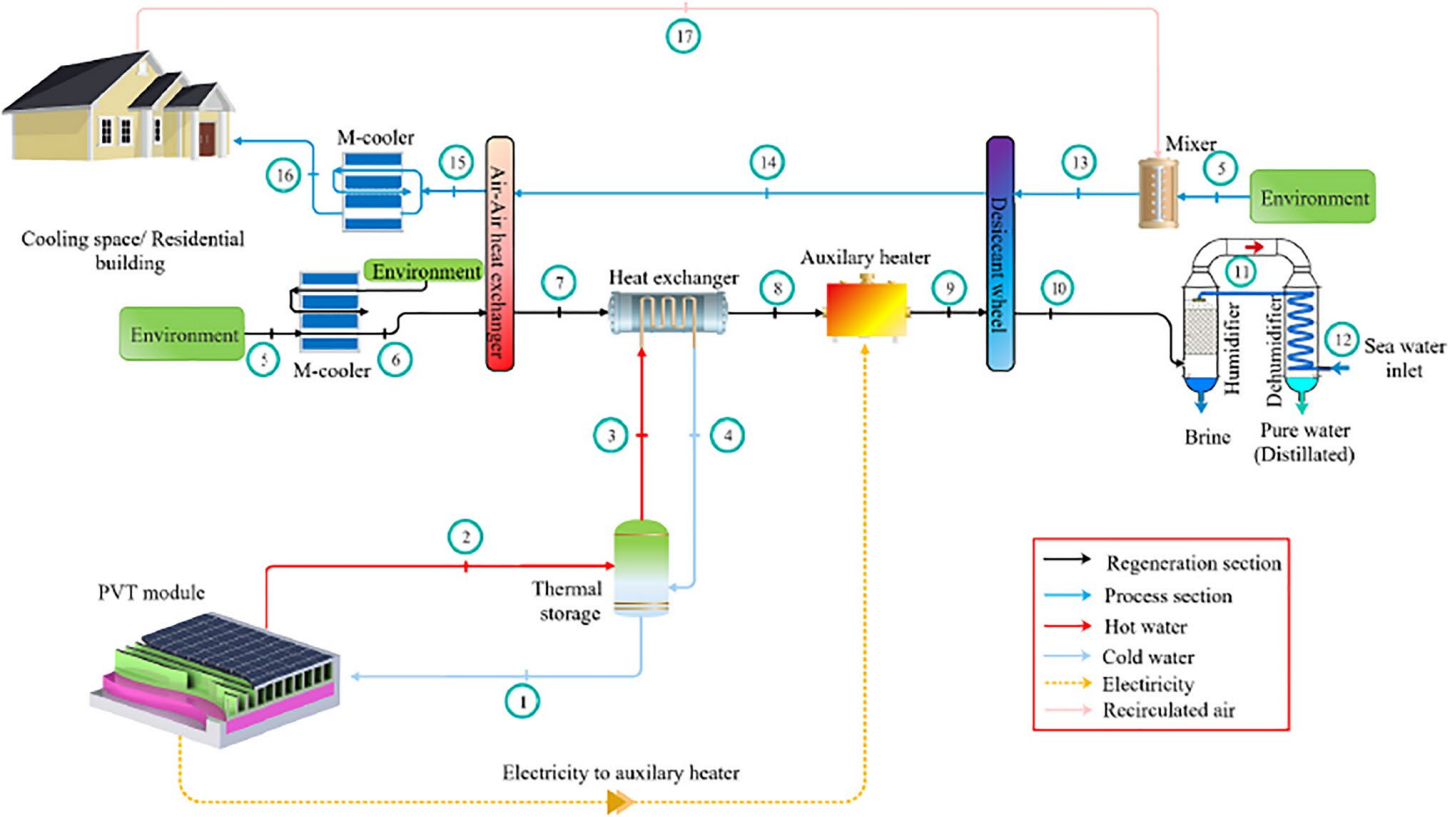
Solar-based desiccant and HDH system (Wang et al. 2020)

**Fig. 21:**
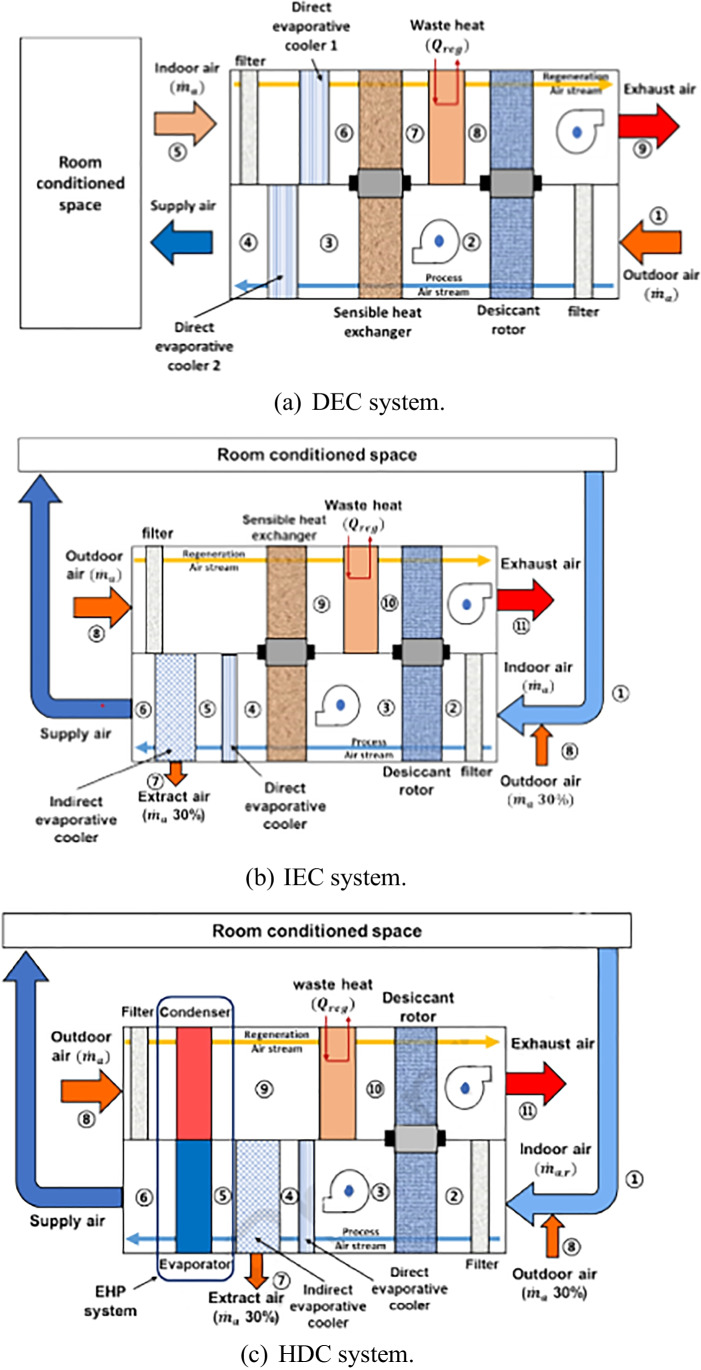
Desiccant cooling systems: **a** DEC system, **b** IEC system, and **c** HDC system (Lee et al. 2021)

**Fig. 22:**
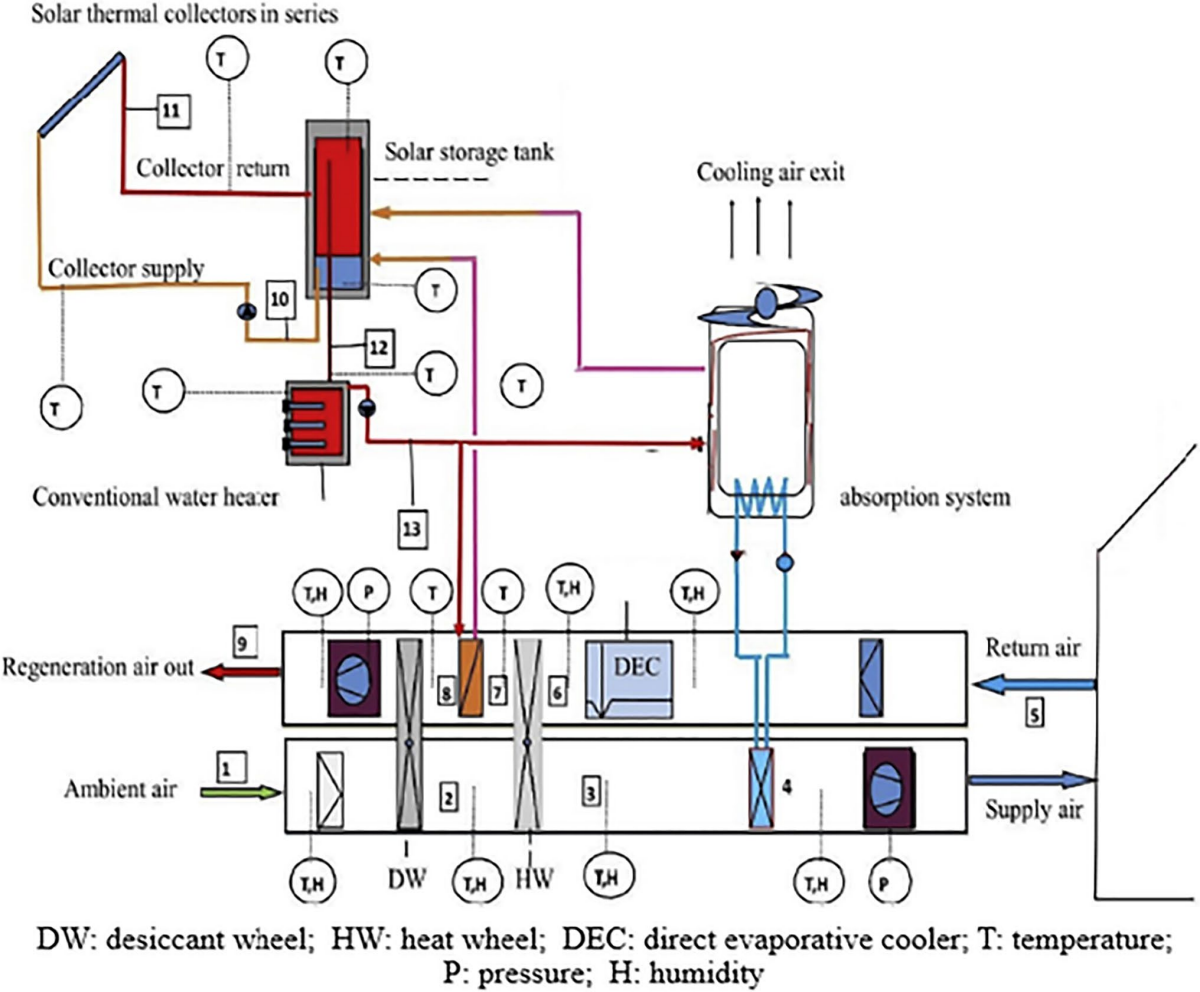
Hybrid absorption-solid desiccant air conditioner (Habib et al. 2020)

**Fig. 23:**
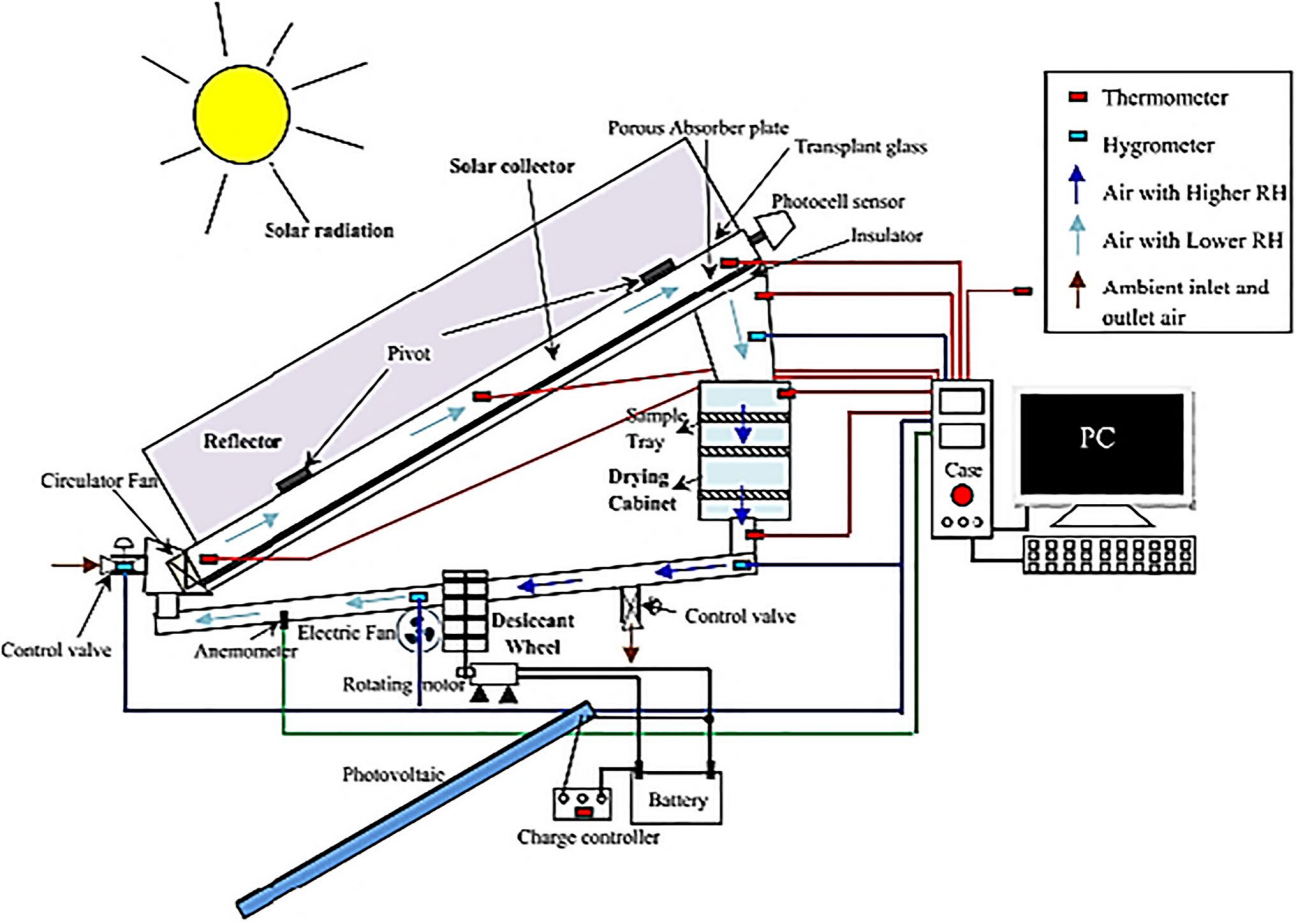
Solar-assisted solid desiccant dryer (Rashidi et al. 2021)

**Fig. 24:**
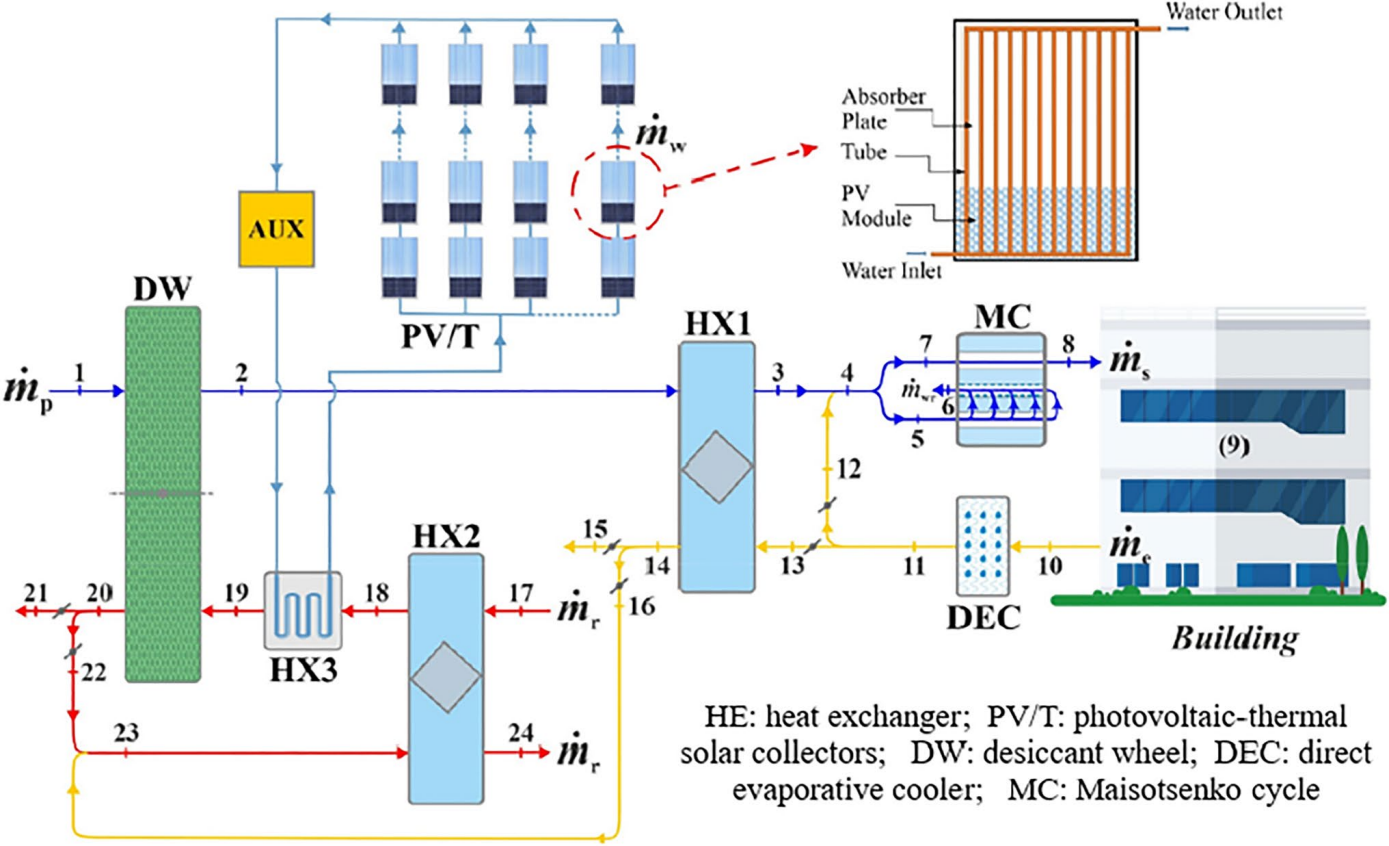
Schematic of desiccant air-conditioning system (Olmuş et al. 2022)

**Fig. 25:**
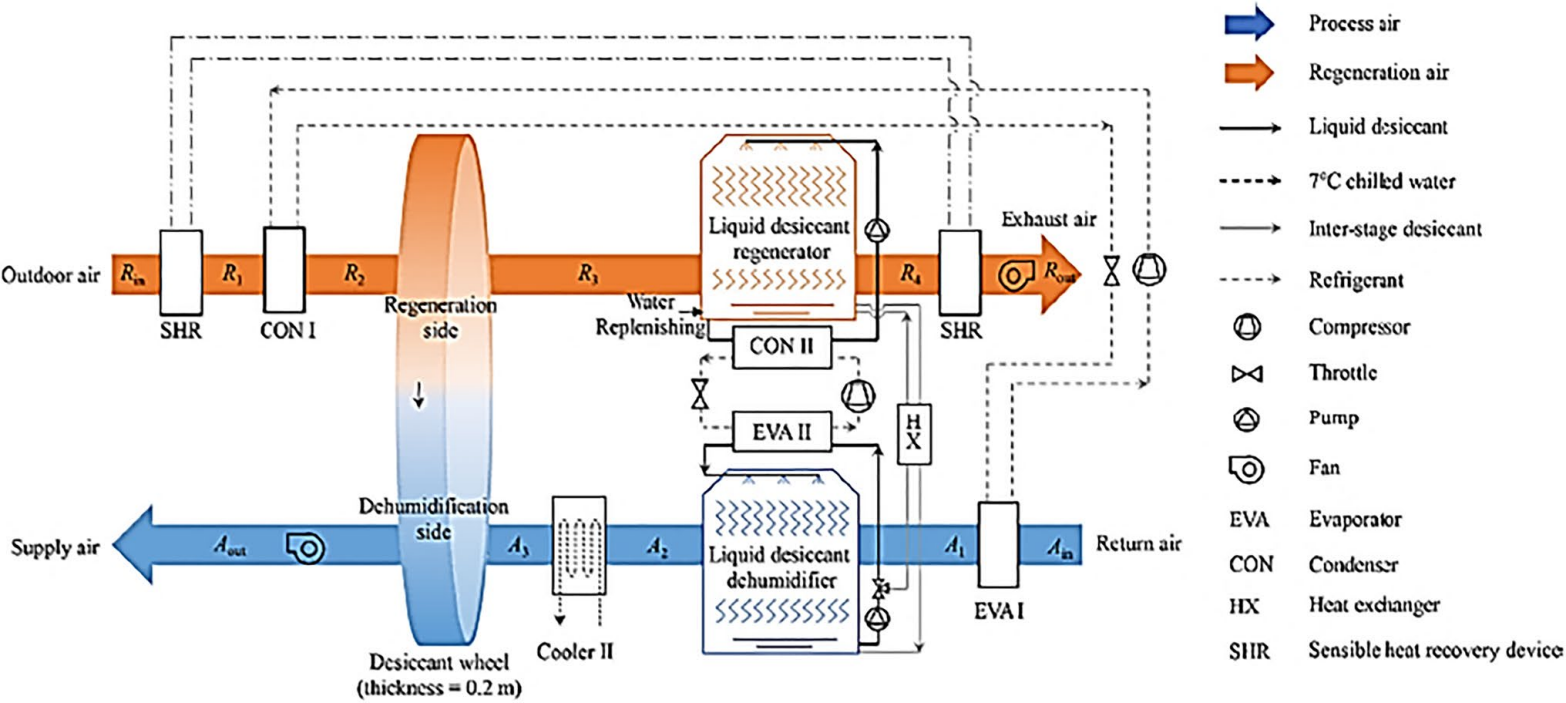
Schematic of conventional air conditioning system for low-humidity environments (Guan et al. 2022)

Different configurations of hybrid desiccant air conditioners are presented to improve a system’s overall performance, for example, the integration of the desiccant air conditioner with a source of renewable energy such as water solar collectors, air solar collectors, and photovoltaic thermal collectors are utilized to heat the regeneration air to reactivate a desiccant material, as a result, the energy consumption decreases and system performance increases. El-Agouz and Kabeel (2014) presented a desiccant system that integrated with an air solar collector and electrical heater as a heat source and geothermal energy for further cooling the process air as shown in Fig. 17, the results confirmed that a supply temperature changed from 12.7 to 21.7 °C for different climate conditions and system performance varied from 0.15 to 1.03. Also, Kabeel and Abdelgaied (2018) conducted a behavior of three configurations of a hybrid system, type A used an electric heater as a heat source, type B, used an air solar collector and electrical air heater, and type C used an air solar collector, phase change material (PCM), and electrical air heater as illustrated in Fig. 18a, b, and c; the results showed that the electricity saving was about 20.85 and 75.82% for type B and type C as compared to type A. Another hybrid system can be introduced to use the highly moist air that leaves desiccant material after the regeneration process to produce pure water, this can be done by integrating a desiccant air conditioner with a desalination unit such as a humidification dehumidification unit (HDH). Abdelgaied et al. (2019) introduced two configurations of a hybrid desiccant integrated with an HDH desalination unit, the difference between them is that the second system utilized an additional solar collector to reheat a regeneration air before entering the desalination unit as shown in Fig. 19a and b, they found that for regeneration flow rates 60 m^3^/h, 120 m^3^/h, 180 m^3^/h, and 240 m^3^/h, the coefficient of performance varied between 0.48–1.11, 0.33–0.61, 0.23–0.42, and 0.18–0.31 and the yield improvement for the second configuration reached 26.5%, 38.96%, 13.16%, and 11.31%, respectively, as compared to first hybrid configuration. Wang et al. (2020) presented a hybrid desiccant system coupled with a photovoltaic thermal solar collector (PVT) and PCM as a heat source and HDH desalination unit as shown in Fig. 20, they concluded that for air flowrates of 0.48, 0.58, 0.68, and 0.78 kg/s, the thermal COP varying between 0.12–0.381, 0.124–0.392, 0.128–0.405, and 0.13–0.411 and freshwater yield varying between 0.76–2.93, 0.86–3.37, 1.02–4, and 1.2–4.9 l/h, respectively. Also, the desiccant air conditioner can be coupled with a vapor-compression or absorption system when a high sensible load exists and in this case, the waste heat from the condenser can utilize to heat a reactivation air required for the reactivation process. Three types of desiccant cooling systems are simulated by Lee et al. (2021) as presented in Fig. 21a, b, and c, the first system based on direct evaporative cooling (DEC), the second based on indirect evaporative cooling (IEC), and the third is hybrid desiccant cooling (HDC) system using DEC, IEC, and vapor compression system. They found that the total performance of HDC was greater than that of IEC when outdoor temperature exceeds 40 °C. Habib et al. (2020) introduced hybrid absorption-solid desiccant air conditioner as presented in Fig. 22, the outcomes showed that the optimized values of coefficient of performance are 0.55 and 1.52 for standalone and integrated absorption systems, respectively. Moreover, the desiccant system can be coupled with a drying unit to preserve different products in supermarkets and stored cereals. Rashidi et al. (2021) conducted a behavior of solar-assisted solid desiccant dryer to dry a single layer of oleaster under different conditions as shown in Fig. 23, they found that the electricity consumption for drying decreased by 11.32–30.15% via using the WRD system. A summary of recent studies on different configurations of hybrid solid desiccant air conditioners is illustrated in Table 1 (Figs. 24 and 25).

**Table 1:** Summary of recent studies on different configurations of hybrid solid desiccant air conditioners

Reference	Type of study	System configuration	Method of regeneration	Remarks	Results
Bourdoukan et al. (2009)	Experimental	Solar desiccant air conditioner	Evacuated tube water solar collector	Desiccant handling unit powered by an Evacuated tube water solar collector The regeneration temperature varied between 55 and 70 °C	The coefficient of performance reached 0.45 for moderately humid climate conditions
El-Agouz and Kabeel (2014)	Simulation using FORTRAN computer program	Solar desiccant air conditioner	Flat plate air solar collector and electrical heater	This system used geothermal energy for further cooling process air before supplying it to the room The regeneration temperature varied between 80 and 120 °C	The outcomes of showed that this hybrid system significantly decreases the temperature of supplied air from 12.7 to 21.7 °C. The supplied air humidity ratio decreased as regeneration temperature increased while it increased as the ambient air humidity increased. The coefficient of performance decreased as the ambient humidity ratio and the regeneration temperature increased and its value ranged between 0.15 and 1.03
Kabeel et al. (2017a)	Numerical	Solar assisted desiccant air conditioner	Air solar collector and electrical heater	The proposed system IDEC with baffle, one for pre-cooling the air inlet to the rotor and another for pre-cooling the air inlet to conditioning space The reactivation temperature varied between 70 and 110 °C	• The optimal working ratio in IDEC with an internal baffle should be 0.15 • Supply temperature increased from 12.7 to 4.2 °C, coefficient of performance increased from 1.55 to 2.36, and supply humidity increased from 76.7 to 77.4%, with an increase in outdoor temperature from 25 to 45 °C • Supply air temperature increased from 12 to 16.6 °C, supply humidity increased from 74.7 to 83.5%, and coefficient of performance increased from 1.28 to 3.34, with an increase in the rate of process air from 220 to 800 m^3^/h • Supply temperature decreased from 15.9 to 10.9 °C, humidity decreased from 82.7 to 71.8%, and coefficient of performance decreased from 3.05 to 1.54, with an increase in reactivation temperature from 70 to 110 °C
Kabeel and Abdelgaied (2018)	Numerical	Solar assisted desiccant air conditioner	Flat plate air solar collector, phase change material (PCM), and electrical heater	Three configurations of solid desiccant air conditioning are presented. Type A used an electrical heater as an energy source, while type B used an air solar collector and electrical air heater and type C used an air solar collector, PCM, and electric air heater For all configurations, the wheel is divided into four parts: two parts for reactivation with an angle of 45° and the others for process with an angle of 135° The reactivation air temperature was kept constant at 80 °C	The results showed that the electricity saving was about 20.85 and 75.82% for type B and type C, compared to type A
Chen and Tan (2020)	Numerical	Solar assisted desiccant air conditioning system	Water solar collectors and electrical heater	This system used high chilled water temperature from cold sources to cool a process air before and after passing over a rotary wheel The reactivation air temperature was kept constant at 80 °C	• The thermal performance of this system was greater than that of conventional solar desiccant air conditioners • The parallel mode was recommended at a supply temperature of 12–18 °C and under low and moderate humidity conditions • The series mode was recommended at a supply temperature between 13.5 and 18 °C and under high humidity conditions
Kabeel et al. (2017b)	Numerical	Hybrid solar-assisted desiccant air conditioner incorporated with HDH desalination units	Double path air solar collector and electrical heater	The reactivation temperature varied between 75 and 95 °C	• The simulated results were validated with published empirical data • By increasing a reactivation temperature from 75 to 95 °C, distillate yields increased from 3.175 to 5.011 L/h, and COP decreased from 4.392 to 3.636 • The distillate yield increased from 2.988 to 4.78 L/h and COP decreased from 4.66 to 3.386 with an increase in a reactivation rate from 70 to 130 m^3^ /h
Abdelgaied et al. (2019)	Experimental	Hybrid solar desiccant air conditioner incorporated with HDH unit	Double path air solar collector + electrical heater	Two configurations of this hybrid system are presented; the difference between them is that a second system utilized an additional solar collector to reheat a reactivation air before entering a desalination unit The reactivation air temperature was kept constant at 85 °C	• COP varied between 0.48–1.11, 0.33–0.61, 0.23–0.42, and 0.18–0.31, and cooling capacity varied between 619.1–860.3, 663.3–900.5, 675.4–924.6, and 687.4–940.7 W, at reactivation flow rate 60 m^3^/h, 120 m^3^/h, 180 m^3^/h, and 240 m^3^/h, respectively • For the second configuration, the yield improvement reached 26.5%, 38.96%, 13.16%, and 11.31%, and the improvement in gain output ratio reached 42.5%, 50.35%, 22.1%, and 18.85% compared to the first configuration at reactivation flow rate 60 m^3^/h, 120 m^3^/h, 180 m^3^/h, and 240 m^3^/h, respectively
Kabeel and Abdelgaied (2019)	Experimental	Hybrid solar assisted desiccant air conditioner incorporated with HDH unit	Double path flat plate air solar collector and electrical heater	In this system, a desiccant dehumidifier with silica-gel baffles and a water cooling cycle for the air conditioner are incorporated with the HDH unit The reactivation air temperature was kept constant at 85 °C	• The new system improved the dehumidification capacity which reached 10.2 g_water_ /kg_dry-air_ (0.3264 g_water_ /g_silica-gel_) • Within a period 9:00 am–6:00 pm the air supply temperature and RH ranged from 17.5 to 18.2 °C and 65–68%, respectively, also the cooling capacity and distillate yield varied between 696–937 W and 3.2–3.6 L /h, respectively • The overall COP varies between 0.40 and 0.622
Wang et al. (2020)	Numerical	Hybrid solar desiccant air conditioner coupled with HDH unit	Photovoltaic/ thermal panels with PCM	• PVT panel represents the heat source to heat a reactivation air and electricity to drive auxiliary heater and fans • PCM represents energy resources at night time The reactivation temperature varied between 68 and 98 °C	• For air flow rates of 0.48, 0.58, 0.68, and 0.78 kg/s, the thermal coefficient of performance changed from 0.12 to 0.381, 0.124–0.392, 0.128–0.405, and 0.13–0.411 and yield changed from 0.76 to 2.93, 0.86–3.37, 1.02–4, and 1.2–4.9 l/h, respectively • The outlet temperature of water from the storage tank was higher than the regeneration air temperature the day due to the use of PCM
Saedpanah and Pasdarshahri (2021)	TRNSYS simulation	Hybrid solar desiccant air conditioner coupled with desalination unit	Water solar collector and PCM	Two modes are presented for providing heating, cooling, and water demand	• Results showed that setting the output humidity at 4.2 g_water_ /kg_dry air_, solar collector area 14.14 m^2^ and TES containing 188.63 kg PCM achieved the best energy, economic, and environmental performance • The results showed that the thermal comfort provided to the demand for heating and cooling increased by 13.9% and the energy consumption decreased significantly over a year. Moreover, the annual yield was 33,619.8 L
Jani et al. (2015)	TRNSYS simulation	Hybrid desiccant air conditioner coupled with vapor compression system	Electrical heater	System based on recirculation mode The reactivation air temperature was kept constant at 94 °C	The latent load was significantly reduced by integrating the desiccant dehumidifier with vapor compression which increased the performance of the system and validated against experimental test data
Jani et al. (2016)	Experimental	Hybrid desiccant air conditioner coupled with vapor compression system	Electrical heater	System based on recirculation mode The reactivation temperature varied between 80 and 140 °C	It is confirmed that a hybrid system is better than a traditional vapor compression under typical humid and hot conditions as it could reduce process air humidity from 18.5 to 7.10 g/kg _dry air_ when it passed to the rotary wheel
Lee et al. (2021)	Simulation using MATLAB	Desiccant air conditioner integrated with direct/indirect evaporative coolers and vapor compression system	Waste heat from condenser and external heat source	Three types of desiccant cooling systems are simulated. The first system is based on direct evaporative cooling (DEC), the second is based on indirect evaporative cooling (IEC) and the third is hybrid desiccant cooling (HDC) using DEC, IEC, and vapor compression systems The reactivation air temperature was kept constant at 70 °C	• Under any operating conditions, the DEC had the lowest performance; it had enough cooling performance as the ambient temperatures not exceeded 35 °C • For outdoor temperatures lower than 40 °C, the coefficient of performance of IEC was higher than that of HDC • At outdoor temperatures exceeding 40 °C, the performance of HDC was higher than IEC
Hussain (2022)	Experimental	Hybrid desiccant air conditioner coupled with vapor compression	Waste heat from condenser and electrical heater	Two hybrid systems are presented, the first regenerated with complete waste heat from the condenser and the other using an electrical heater The reactivation temperature varied between 60 and 70 °C	System performance, electric coefficient of performance, and dehumidification effectiveness for both systems decreased with increased process inlet temperature at different process velocities and constant reactivation velocity at 2.5 m/s
Habib et al. (2020)	TRNSYS and GenOpt simulation	Hybrid absorption-adsorption air conditioning system	Water solar collector	The proposed system consists of three sub-systems, desiccant dehumidification system, solar heating system, and absorption chiller The reactivation air temperature was kept constant at 70 °C	The resulting optimized values of the thermal coefficient of performance are 1.52 and 0.55 while the optimized value of solar fraction reached 57.50% and 56.25% for standalone and integrated absorption systems, respectively
Ali et al. (2022)	Experimental	Hybrid absorption- solid desiccant air conditioner	Electrical heater	The experimental system consists of a gas-fired air-cooled NH_3_-H_2_O absorption chiller to handle the sensible load and a silica-gel-based solid desiccant for handling the latent load of the space The reactivation air temperature was kept constant at 80 °C	• The hybrid system cooling capacity was about 2 KW higher than the conventional solid desiccant system • The hybrid system COP was 50–55% higher than the conventional system and also as compared with the double-effect absorption chiller
Misha et al. (2016)	Experimental	Solar-assisted solid desiccant dryer	Water solar collector + electrical heater		• The drying time of the products was reduced by 64%, 44%, and 33%, for the first, second, and third columns, respectively, compared with open sun drying that required 30 h and 40 min. to reduce the moisture of the product from 69 to 29% • At full capacity, the drying efficiency was 19% and the drying rate was 8.37 kg/h
Rashidi et al. (2021)	Experimental	Solar-assisted solid desiccant dryer		Different dryer conditions were considered to dry a single layer: with reflectors (WR), with reflectors + desiccant (WRD), and without reflectors + desiccant (WORD) The maximum drying temperature for WR, WRD, and WORD varies from 68.1 to 74.1 °C, 67.4–70.0 °C, and 66.9–69.9 °C, respectively	• The power consumption for oleaster drying decreased by 11.32–30.15% via using the WRD system • For the WRD system, the overall efficiency was ranged from 33.21 to 39.64% which higher than the WORD system
Güzelel et al. (2022)	Theoretically	Rotary desiccant air conditioners integrated with heat recovery units, dew-point indirect evaporative coolers, and direct evaporative coolers	Electrical heater	The desired comfort conditions could be obtained for the building during the complete cooling season The reactivation air temperature was kept constant at 70 °C	The average monthly thermal COP reached a maximum value of 0.78 in October and a minimum value of 0.22 in July
Olmuş et al. (2022)	Theoretically	Photovoltaic-thermal solar collectors powered the rotary desiccant air conditioners integrated with heat recovery units, dew-point indirect evaporative coolers, and direct evaporative coolers (Fig. 24)	Photovoltaic-thermal solar collectors and electrical heater	The reactivation air temperature was kept constant at 70 °C	The thermal coefficient of performance varies between 0.28 and 0.40 during the day
Guan et al. (2022)	Theoretically	Conventional air conditioning systems are suitable for low-humidity environments (Fig. 25)	Heat recovery device and condenser	Innovatively incorporated solid desiccant and liquid desiccant with conventional air conditioners. The reactivation temperature varied between 51.5 and 81.6 °C	The results presented that COP reached 1.6 and the electricity consumption was reduced by a rate varying between 19.6 and 58.0% during the summer
Chen and Shi (2022)	Experimental	Closed-cycle rotary desiccant air conditioner	Electrical heater	The reactivation air temperature was kept constant at 80 °C	The results showed that the exergy efficiency reached 50.05%

## Conclusion

Recently, due to the many advantages of desiccant air conditioners (no use of ozone-depleting refrigerants, highly efficient moisture control, etc.) compared to the traditional vapor-compression refrigeration system. Thus, desiccant air conditioners are attracting increasing interest from researchers. Several studies have been conducted primarily aiming at improving the overall performance of desiccant air conditioners by innovating new composite desiccant materials, innovating new system configurations and improving system designs and controls, and integrating different hybrid energy systems/technologies. This comprehensive review focuses on the new innovative desiccant materials, the most important composite desiccant materials, the most important new innovative designs and configurations of desiccant air conditioners and system controls, and the most important hybrid energy sub-systems/technologies integrated with desiccant air conditioners. The most important findings of the comprehensive review are as follows:

- The use of geothermal energy to cool the supply air before it enters a conditioning room for solar-assisted desiccant air conditioners, leading to a decrease in the temperature of supplying air at an entrance to a conditioning room to 12.7 °C, as well as improved the overall coefficient of performance to 1.03.
- The use of an IDEC with baffles to cool the supply air before it enters to conditioning room for solar-assisted hybrid rotary desiccant air conditioners. This led to increasing the supply temperature from 12.7 to 14.2 °C, increasing the coefficient of performance from 1.55 to 2.36, and increasing the supply humidity from 76.7 to 77.4% with an increase in the reactivation temperature from 70 to 110 °C.
- An added energy storage material leads to an increase in the saving in electrical power consumption in solar-assisted hybrid rotary desiccant air conditioners to 75.82%.
- The combination of the HDH desalination unit and solar collector with the solar-assisted hybrid rotary desiccant air conditioners represents a good choice to achieve the highest overall performance. By increasing the reactivation temperature from 75 to 95 °C, the distillate yield increased from 3.175 to 5.011 L/h and the overall COP decreased from 4.392 to 3.636.
- The combination of solar-assisted hybrid rotary desiccant air conditioners with a vapor compression system reduced the humidity of process air from 18.5 to 7.10 g/kg_dry air_.

### Recommendation and future scope

Based on the comprehensive review presented above, we presented some discussions on issues and recommendations for future work that can help focus the necessary efforts to find solutions to urgent and pending problems, which lead to further improvements in the overall performance of desiccant air conditioners as follows:

- The discontinuity of solar energy represents one of the main challenges facing the incorporated solar energy with desiccant air conditioning. To treat intermittent solar energy, energy storage materials are used, where part of the thermal energy is stored in periods of high intensity of solar energy, and this energy is recovered again in periods of low solar energy intensity and the period after sunset.
- Solar concentrators that contain energy storage materials must be integrated with the desiccant air conditioning system to achieve the highest benefit rates from solar energy in improving the overall system performance.
- More research is needed to develop strategies of optimization control for solar-desiccant air conditioners, considering the dynamic performance of energy storage materials as well as the uncertainties of the optimal control strategy.
- More research is needed to develop innovative new types of composite desiccant materials that have high thermal properties.
- Innovating a hybrid system that combines desiccant air conditioning, PVT panels, solar concentrator, and HDH desalination unit, suitable for remote regions to achieve thermal comfort conditions, electricity generation, and produce fresh water.
- Innovating a hybrid system that combines desiccant air conditioning, PVT panels, solar concentrator, and drying unit, suitable for remote regions to achieve thermal comfort conditions, electricity generation, and drying plants.

## Data Availability

Not applicable

## Abbreviations

COP: Cofficient of performance
DEC: Direct evaporative cooler
HDC: Hybrid desiccant cooling
HDH: Humidification-dehumidification desalination
HVAC: Heating, ventilation, and air conditioning
IEC: Indirect evaporative cooler
OTSDC: One-rotor two-stage desiccant cooling
PCM: Phase change materials
PVT: Photovoltaic thermal solar collector
TSDC: Two-stage desiccant cooling
VRF: Variable refrigerant flow
